# Phosphorylation of 14-3-3ζ links YAP transcriptional activation to hypoxic glycolysis for tumorigenesis

**DOI:** 10.1038/s41389-019-0143-1

**Published:** 2019-05-10

**Authors:** Yu Jia, Hui-Yan Li, Jue Wang, Ying Wang, Peng Zhang, Ning Ma, Shi-Jing Mo

**Affiliations:** 1 Cancer Research Center, Tongji Hospital, Tongji Medical College, Huazhong University of Science and Technology, 430030 Wuhan, Hubei People’s Republic of China; 2 General Surgical Laboratory, The First Affiliated Hospital, Sun Yat-Sen University, 510080 Guangzhou, Guangdong People’s Republic of China; 3 Department of Pathology, The First Affiliated Hospital, Sun Yet-Sen University, 510080 Guangzhou, Guangdong People’s Republic of China; 4 Department of Surgery, Huashan Hospital, Fudan University, 200040 Shanghai, People’s Republic of China; 5 Department of Hepatobiliary Surgery II, Zhujiang Hospital, Southern Medical University, 510280 Guangzhou, Guangdong People’s Republic of China

**Keywords:** Cancer metabolism, Phosphorylation

## Abstract

Hypoxic microenvironment deregulates metabolic homeostasis in cancer cells albeit the underlying mechanisms involved in this process remain hitherto enigmatic. 14-3-3ζ/Yes-associated protein (YAP) axis plays a principal role in malignant transformation and tumor development. Here, we report that hypoxia disassembles 14-3-3ζ from YAP and thereby promotes YAP nuclear localization mediated by ERK2, which directly binds to the D-site of mitogen-activated protein kinase (MAPK) docking domain in 14-3-3ζ Leu98/100 and phosphorylates 14-3-3ζ at Ser37. When localizing in nucleus, YAP recruits at pyruvate kinase M2 (PKM2) gene promoter with hypoxia-inducible factor 1α (HIF-1α), for which PKM2 transcription is required. 14-3-3ζ Ser37 phosphorylation is instrumental for the hypoxia-induced glucose uptake, lactate production, and clonogenicity of pancreatic ductal adenocarcinoma (PDAC) cells, as well as tumorigenesis in mice. The 14-3-3ζ Ser37 phosphorylation positively correlates with p-ERK1/2 activity and HIF-1α expression in clinical samples from patients with PDAC and predicts unfavorable prognosis. Our findings underscore an appreciable linkage between YAP transcriptional activation and hypoxic glycolysis governed by ERK2-dependent 14-3-3ζ Ser37 phosphorylation for malignant progression of PDAC.

## Introduction

Rapid and reversible stress response is necessary for metabolic homeostasis, which satisfies the demands of tumor growth in the face of a dynamically altering microenvironment. Unlike non-tumorigenic tissues, cancer lesions often display excessive glucose uptake, redundant lactate production, and bare oxygen consumption, a phenomenon defined as glycolysis^1,2^. In order to maintain distinct malignant features, cancer cells frequently modulate themselves to undergo metabolic reprogramming for adapting to hypoxic microenvironment. Hypoxia-inducible factor 1α (HIF-1α), a subunit of hypoxia-responsive HIF-1 transcription factor (TF), is implicated in the pathogenesis of cancer initiation and progression. Under hypoxic circumstance, HIF-1α dimerizes with HIF-1β or other TFs in nucleus to occupy in hypoxia response element (HRE) within the promoter of pyruvate kinase M2 (PKM2) gene and enhance its transcription, which then contributes to glycolysis and tumorigenesis^3^.

In mammalian cells, yes-associated protein (YAP) has been identified as a pleiotropic transcriptional co-activator that participates in numerous biological processes, ranging from tissue differentiation, apico-basolateral polarity, mitosis to notably, tumor development^4,5^. Interestingly, the transcriptional activity of YAP in cancer cells is dependent on aerobic glycolysis^6^. Increasing evidence show that deregulation of redox homeostasis mediated by HIF-2α results in increased steady-state levels of YAP in cancer cells^7^, and that HIF‐1/2α is capable of enhancing G protein-coupled receptor GPRC5A transcription, which leads to YAP activation under hypoxic conditions^8^. To date, the subcellular redistribution of YAP secondarily as a consequence of LATS1/2 protease activation originated from Hippo pathway in response to hypoxic stress has been documented. Hypoxia destabilizes LATS1/2 through E3 ligase seven in absentia homolog (SIAH2), thereby promoting YAP, together with its paralog WWTR1 (WW domain-containing transcription regulator protein 1, known as TAZ), to translocate into nucleus, where they interact with the TEAD4 protein to transactivate multiple downstream targets^9,10^. Besides SIAH2, phosphorylation at Serine (Ser)128 by nemo-like kinase (NLK) is able to promote YAP nuclear localization and the casein kinase 1 (CK1)-induced Ser384 phosphorylation also disrupts cytoplasmic YAP inclusion^11,12^. By contrary, 14-3-3, a highly conserved, acidic proteins expressing ubiquitously in eukaryotes, evokes cytoplasmic YAP sequestration, which in turn prevents further signal amplification^13^. Seven isoforms of 14-3-3 family have been described in mammals, including β, ε, η, γ, τ, ζ, and σ. Human 14-3-3ζ molecule is comprised of nine antiparallel α-helices with an amphipathic ligand-binding groove^14^. This property enables it to assemble with ligands of different sizes and shapes, expanding the repertoire of its significance in signal transduction to a variety of cellular responses including self-renewal, survival, metabolism, and growth. 14-3-3ζ plays an important role in malignant transformation, while its dysregulation is linked to oncogenesis and recurrence of human cancers^15–17^. However, the intrinsic nature controlling subcellular redistribution of YAP during hypoxia remains poorly understood and much need to be uncovered regarding the potential role of 14-3-3ζ in tumor development.

In the present study, we report that ERK2 binds to the D-site of mitogen-activated protein kinase (MAPK) docking domain in 14-3-3ζ Leu98/100 and phosphorylates 14-3-3ζ at Ser37 in response to hypoxia. This event leads to disassembly of 14-3-3ζ from YAP and subsequently promotes YAP nuclear localization. When localizing in nucleus, YAP cooperates with HIF-1α to transactivate PKM2, thus augmenting glucose uptake, lactate production, and tumorigenesis of pancreatic ductal adenocarcinoma (PDAC) cell lines. The 14-3-3ζ Ser37 phosphorylation positively correlates with p-ERK1/2 activity and HIF-1α expression in clinical samples from PDAC patients and predicts unfavorable prognosis. Our study provide a molecular rationale for an unappreciated role of ERK2-dependent 14-3-3ζ phosphorylation in connecting YAP transcriptional activation with hypoxic glycolysis during PDAC tumorigenesis.

## Results

### Hypoxia disassembles 14-3-3ζ from YAP and promotes nuclear localization of YAP

**Fig. 1:**
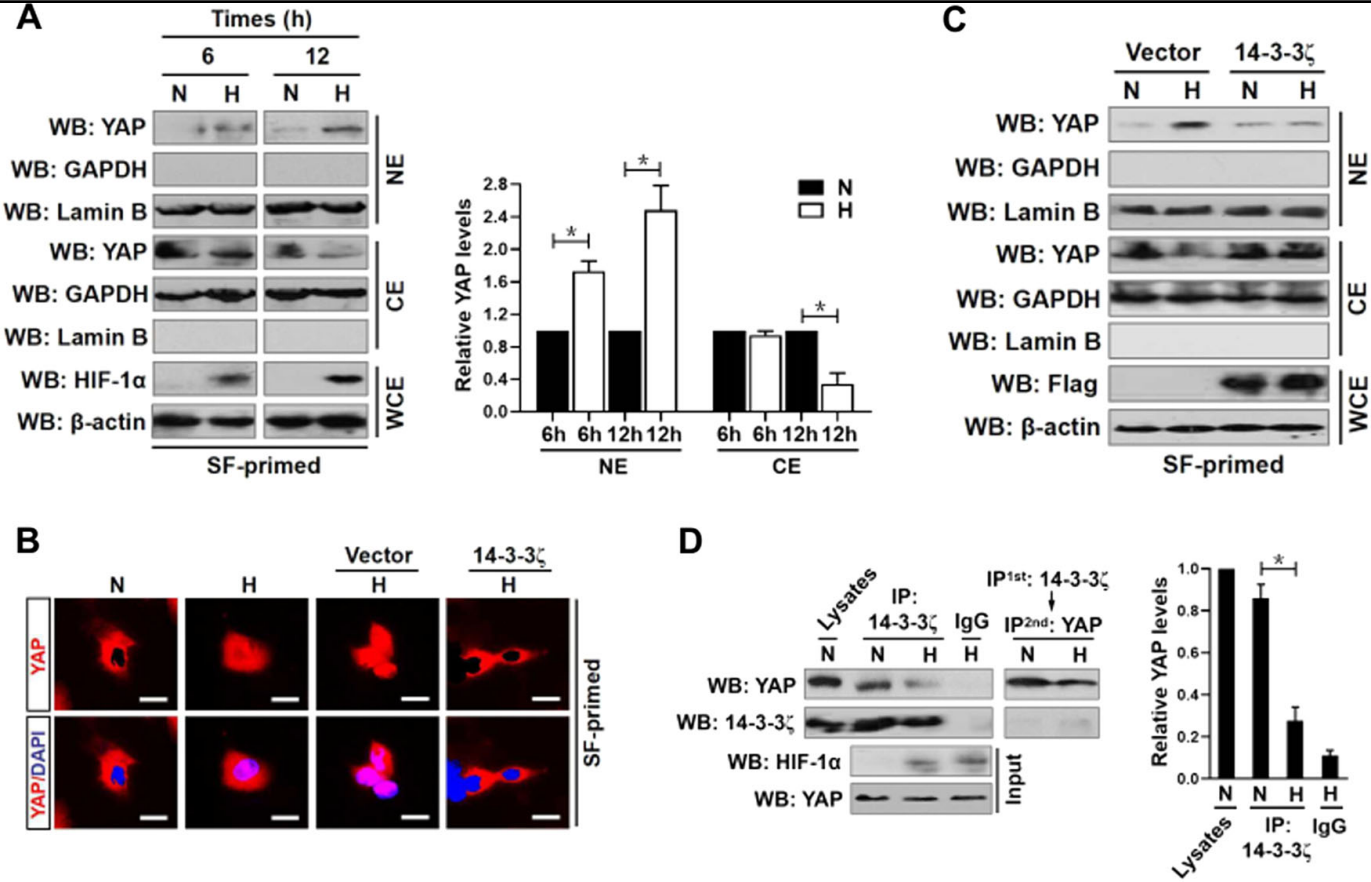
Hypoxia disassembles 14-3-3ζ from YAP and promotes nuclear localization of YAP. **a** Subcellular fractionation analyses examining abundance of nuclear and cytoplasmic YAP protein expression in SW-1990 PDAC cells stimulated with hypoxia in the indicated times. GAPDH and Lamin B were used as internal control of cytoplasmic and nuclear extractions, respectively. SF serum-free, N normoxia, H hypoxia, WB western-blotting, NE nuclear extraction, CE cytoplasmic extraction, WCE whole cell extraction. Data are expressed as mean ± s.d. of three independent experiments. *P < 0.05. Two-sided Student’s *t* test was used to calculate the *P* value. **b** Representative immunfluorescence images of nuclear YAP localization in SW-1990 PDAC cells stimulated with hypoxia in the presence or absence of Flag-tagged wild-type 14-3-3ζ transfection for 6 h. Scale bar = 25 μm. **c** Subcellular fractionation analyses detecting abundance of nuclear and cytoplasmic YAP protein expression in hypoxia-stimulated SW-1990 PDAC cells with or without Flag-tagged wild-type 14-3-3ζ transfection. **d** Coimmunoprecipitation assay evaluating the interaction between 14-3-3ζ and YAP in SW-1990 PDAC cells stimulated with hypoxia for 6 h. Data are expressed as mean ± s.d. of three independent experiments. *P < 0.05. Two-sided ANOVA with Bonferroni post hoc *t* test correction was used to calculate the *P* value

YAP is a pleiotropic TF that can shuttles from cytoplasm into nucleus in response to environmental stress^18^. However, the relationship between hypoxia and YAP during tumorigenesis is incompletely understood. We first examined whether hypoxia influences YAP function in a subcellular-compartment-dependent fashion. For this purpose, we primed SW-1990 PDAC cells (S-1) under serum-free (SF) mediums and then stimulated them with hypoxia over different periods of time. Subcellular fractionation analyses showed that YAP predominantly presented in the cytoplasmic fractions under normoxic conditions, and stimulation of S-1 cells with hypoxia caused a time-dependent increase in the steady-state levels of nuclear YAP while concomitantly led to a notable reduction in cytoplasmic YAP (Fig. 1a). We did not observe any glyceraldehyde 3-phosphate dehydrogenase (GAPDH) in the nuclear fractions or Lamin B in the cytoplasmic fractions, suggesting that the subcellular fractionation approach is free of cross-contamination. The hypoxia-stimulated YAP nuclear accumulation is not restricted to PDAC cells since such response could be recapitulated in A498 renal carcinoma (RCC) cells and Huh-7 hepatocellular carcinoma (HCC) cells (Supplemental Fig. S1a). These data are consistent with the immunofluorescence (IF) findings that YAP substantially translocated into nucleus following hypoxia stimuli (Fig. 1b). Hypoxia-mimetic agent cobalt chloride (CoCl_2_) also stimulated YAP nuclear translocation in S-1 cells, as judged by the result that nuclear YAP was weak at initial but gradually appeared and built up beyond 0.1 mmol/L CoCl_2_ treatment (Supplemental Fig. S1b). Depletion of HIF-1α with small interfering RNA (siRNA) targeting HIF-1α had minimal effect on the hypoxia-stimulated YAP nuclear translocation (Supplemental Fig. S1c), indicating a HIF-1α-independent mechanism may be engaged. To test whether YAP binds to TEAD4 when translocated into nucleus, we performed co-immunoprecipitation (co-IP) assay in nuclear fractions from the hypoxia-stimulated S-1 cells expressing Myc-tagged wild-type TEAD4. Western-blotting (WB) of the immunoprecipitated Myc with an anti-YAP antibody corroborated that hypoxia stimulated an interaction between Myc-tagged TEAD4 and YAP without significantly affecting the stabilization of Myc-TEAD4 (Supplemental Fig. S1d). The immunoprecipitated immunoglobulin G (IgG) as the control did not contain any proteins under all experimental duration. These data strongly demonstrate that hypoxia induces the nuclear localization of YAP in several tumor cell lines including human PDAC.

Nuclear import/export of YAP is tightly balanced by 14-3-3ζ, as evidenced by the fact that 14-3-3ζ assembles with YAP, sequesters it in the cytoplasm and prevents it from transactivating target genes^15^. Indeed, the siRNA-mediated silencing of endogenous 14-3-3ζ (termed as e14-3-3ζ hereafter) increased the amount of YAP in nucleus (Supplemental Fig. S2a), which is equivalent to that caused by hypoxia. In stark contrast, a dramatic diminution in nuclear YAP accumulation was observed after ectopic expression of the Flag-tagged wild-type 14-3-3ζ in hypoxia-stimulated S-1 cells (Fig. 1b, c). Co-IP assay of nuclear fractions from the hypoxia-stimulated S-1 cells harboring Flag-tagged wild-type 14-3-3ζ identified much less YAP in the immunoprecipitates (IPs) of Myc-TEAD4 when compared with the cells harboring empty vector (Supplemental Fig. S2b). Taken together, 14-3-3ζ blocks nuclear localization of YAP under hypoxic circumstances.

The ability of 14-3-3ζ to block YAP nuclear localization under hypoxia and the exclusive sequestration of YAP by 14-3-3ζ prompted us to pursue the hypothesis that hypoxia promotes nuclear YAP localization through disassembling 14-3-3ζ from YAP. To approach this, we explored the interaction between 14-3-3ζ and YAP using co-IP assay before and after hypoxia stimuli. WB of immunoprecipitated e14-3-3ζ with an anti-YAP antibody revealed that hypoxia profoundly reduced the abundance of YAP in IPs of e14-3-3ζ (Fig. 1d), which conversely correlated with the increased YAP nuclear accumulation under the same conditions (Fig. 1a, b). Consistent with these results, CoCl_2_ treatment blunted 14-3-3ζ-YAP interaction as efficiently as hypoxia stimuli did (Supplemental Fig. S2c). Nevertheless, hypoxia had no impact on the interaction between YAP and WWTR1, the paralog of YAP (Supplemental Fig. S2d), suggesting that the observed disassembly of 14-3-3ζ from YAP is probably due to a posttranslational modification on 14-3-3ζ by hypoxia. WWTR1 also binds to 14-3-3ζ (Supplemental Fig. S2e), and hypoxia stimuli was sufficient to block their interaction, supporting the notion that 14-3-3ζ may disassociate from the YAP/TAZ complex upon hypoxia. Collectively, these results indicate that hypoxia disassembles 14-3-3ζ from YAP, thereby promoting YAP nuclear localization.

### ERK2 is required for the hypoxia-induced disassembly of 14-3-3ζ from YAP and nuclear YAP localization

**Fig. 2:**
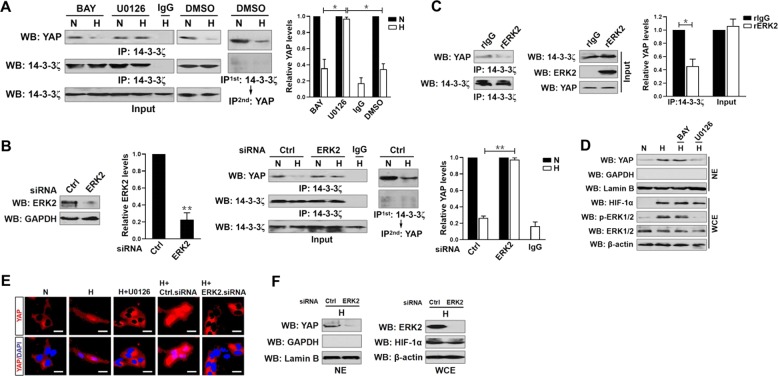
ERK2 is required for the hypoxia-induced disassembly of 14-3-3ζ from YAP and nuclear YAP localization. **a** Coimmunoprecipitation assay assessing the interaction between 14-3-3ζ and YAP in SW-1990 PDAC cells stimulated with hypoxia in the presence or absence of 5 μmol/L BAY 11-7085 (BAY) and 20 μmol/L U0126 pretreatment. Data are expressed as mean ± s.d. of three independent experiments. *P < 0.05. Two-sided ANOVA with Bonferroni post hoc *t* test correction was used to calculate the *P* value. **b** Left panel: Western-blotting assessing the levels of ERK2 expression in SW-1990 PDAC cells transfected with ERK2 siRNA. Data are expressed as mean ± s.d. of three independent experiments. **P < 0.01. Two-sided Student’s *t* test was used to calculate the *P* value. Right panel: Coimmunoprecipitation assay detecting the interaction between 14-3-3ζ and YAP in SW-1990 PDAC cells stimulated with hypoxia in the presence or absence of ERK2 siRNA transfection. Data are expressed as mean ± s.d. of three independent experiments. **P < 0.01. Two-sided ANOVA with Bonferroni post hoc *t* test correction was used to calculate the *P* value. **c** In vitro protein binding assay with mixing recombinant ERK2 (rERK2) kinase and IPs of e14-3-3ζ from SW-1990 PDAC cells followed by WB analyses with an anti-YAP antibody. Data are expressed as mean ± s.d. of three independent experiments. *P < 0.05. Two-sided Student’s *t* test was used to calculate the *P* value. **d** Subcellular fractionation analyses assessing nuclear YAP localization in hypoxia-stimulated SW-1990 PDAC cells with 5 μmol/L BAY 11-7085 (BAY) and 20 μmol/L U0126 pretreatment, respectively. **e** Representative immunfluorescence images of nuclear YAP localization in hypoxia-stimulated SW-1990 PDAC cells with 5 μmol/L BAY 11-7085 (BAY) and 20 μmol/L U0126 pretreatment, respectively, and in hypoxia-stimulated SW-1990 PDAC cells with or without ERK2 siRNA transfection. Scale bar = 25 μm. **f** Subcellular fractionation analyses comparing nuclear YAP localization in hypoxia-stimulated SW-1990 PDAC cells with or without ERK2 siRNA transfection

Hypoxic stress is known to activate numerous oncogenic signaling cascades such as NF-κB and MEK/ERK^19,20^. We investigated the molecular mechanism whereby hypoxia disassembles 14-3-3ζ from YAP by pretreating the hypoxia-stimulated S-1 cells with NF-κB pathway inhibitor BAY 11-7085 or MEK kinase inhibitor U0126 that impaired the ability of hypoxia to induce IκBα (Supplemental Fig. S3a, top panel) and ERK1/2 (Supplemental Fig. S3a, bottom panel) phosphorylation, respectively. Dephosphorylation of ERK1/2, but not that of IκBα, abrogated the hypoxia-stimulated disassembly of 14-3-3ζ from YAP, as the hypoxia-declined YAP abundance in IPs of e14-3-3ζ was almost entirely restored by U0126 but was barely affected by BAY 11-7085 pretreatment (Fig. 2a). The requirement of ERK for the hypoxia-stimulated disassembly of 14-3-3ζ from YAP was further confirmed by the ERK2 siRNA-transfected cells, which displayed increased YAP abundance in immunoprecipitated e14-3-3ζ in contrast to the cells transfected with control siRNA under hypoxia (Fig. 2b). Coincide with the results described for the pharmacological and genetic blockade of 14-3-3ζ-YAP disassembly upon hypoxia, in vitro kinase assays with mixing recombinant ERK2 (rERK2) kinase and IPs of e14-3-3ζ followed by WB analyses identified a great impediment for 14-3-3ζ-YAP binding in the presence of ERK2 (Fig. 2c). These data imply that ERK2 is essential for the hypoxia-stimulated disassembly of 14-3-3 ζ from YAP in PDAC cells.

We noticed that cells with hypoxia single stimuli or hypoxia plus BAY 11-7085 costimuli had increased YAP nuclear accumulation in comparison to the cells without, whereas the cells that were costimulated with hypoxia plus U0126 did not (Fig. 2d, e). Unlike the parental cells bearing control siRNA, the cells bearing ERK2 siRNA displayed lack of nuclear YAP accumulation during the whole course of deoxygenated experiments (Fig. 2e, f), accompanied by the prominent downregulation of total ERK2. Previous reports demonstrated that serum stimuli (SS) enhances transcriptional activity of YAP via triggering nuclear translocation of YAP^21^. As shown in Supplemental Fig. S3b, the SS-triggered interaction between nuclear YAP and Myc-TEAD4 was intact in the ERK2 siRNA-beared cells when compared with the control cells, suggesting that SS triggers YAP nuclear localization via a mechanism distinct from that induced by hypoxia.

### ERK2 directly interacts with and phosphorylates 14-3-3ζ at Ser37 in response to hypoxia

**Fig. 3:**
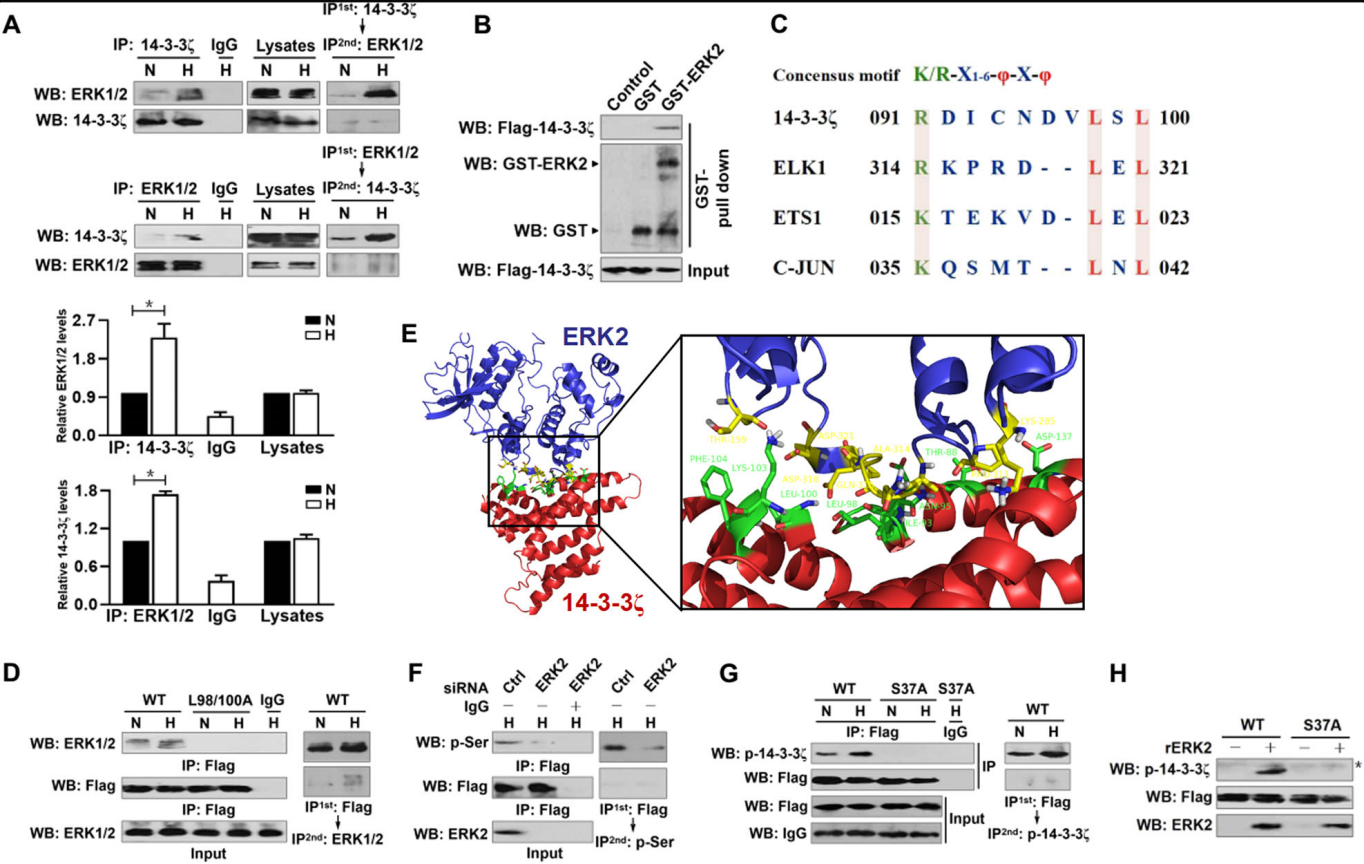
ERK2 directly interacts with and phosphorylates 14-3-3ζ at Ser37 in response to hypoxia. **a** Coimmunoprecipitation assay determining the interaction between endogenous 14-3-3ζ and ERK1/2 in SW-1990 PDAC cells stimulated with hypoxia for 6 h. Data are expressed as mean ± s.d. of three independent experiments. *P < 0.05. Two-sided Student’s t test was used to calculate the *P* value. **b** GST pull-down assay examining the interaction between purified GST-ERK2 proteins and IPs of Flag-tagged wild-type 14-3-3ζ from hypoxia-stimulated SW-1990 PDAC cells. **c** Recognition D-site motif of 14-3-3ζ and other known ERK2 substrates for comparison. **d** Coimmunoprecipitation assay determining the interaction between Flag-tagged wild-type 14-3-3ζ or mutant 14-3-3ζ L98/100 A and ERK1/2 in SW-1990 PDAC cells stimulated with hypoxia for 6 h. **e** Structural model of the ERK2 (PDB ID: 4H3Q) and 14-3-3ζ (PDB ID: 1IB1) complex. The interacting residues involved in the interface between ERK2 and D-site peptide of mitogen-activated protein kinase (MAPK) docking domain at 14-3-3ζ are as indicated. **f** Coimmunoprecipitation assay assessing the abundance of Flag-tagged wild-type 14-3-3ζ phosphorylation in hypoxia-stimulated SW-1990 PDAC cells in the presence or absence of ERK2 siRNA transfection with an anti-phospho-serine antibody. **g** Coimmunoprecipitation assay determining the levels of Flag-tagged wild-type 14-3-3ζ (WT) and mutant 14-3-3ζ S37A (S37A) phosphorylation in hypoxia-stimulated SW-1990 PDAC cells with an anti-14-3-3ζ pS37 antibody. **h** In vitro protein kinase assay for detection of 14-3-3ζ Ser37 phosphorylation with mixing recombinant ERK2 protein (rERK2) and IPs of Flag-tagged wild-type 14-3-3ζ (WT) or mutant 14-3-3ζ S37A (S37A) followed by WB analyses with an anti-14-3-3ζ pS37 antibody. Asterisk indicates the non-specific bands

To clarify whether 14-3-3ζ has a crucial role in the ERK2-initiated disassembly of 14-3-3ζ from YAP under hypoxia, we western-blotted the e14-3-3ζ immobilizing in agarose beads with an anti-ERK1/2 antibody and observed that hypoxia stimuli resulted in ERK1/2 binding to e14-3-3ζ (Fig. 3a, top panel). Reciprocally, 14-3-3ζ was detected in IPs of endogenous ERK1/2, and this was further enhanced by hypoxia (Fig. 3a, bottom panel). An in vitro glutathione S-transferase (GST) pulldown assay with mixing purified GST-ERK2 and IPs of Flag-tagged wild-type 14-3-3ζ from hypoxia-stimulated cells also showed that the two proteins interacted directly (Fig. 3b). MAPKs bind to their substrates via a docking groove comprised of acidic common docking domain and glutamic acid-aspartic acid pocket^22^. ERK substrates mostly share a consensus MAPK docking D-site consisting of hydrophobic (φ)-X-hydrophobic (φ) motif (K/R-X_1–6_-φ-X-φ)^23,24^. The 14-3-3ζ amino acid sequence contains a D-site peptide 91-RDICNDVLSL-100, which exhibits optimal match with the MAPK docking D-site as other ERK substrates do (Fig. 3c). Of note, the D-site mutant, in which Leucine98/100 (L98/100) were mutated to alanine (A98/100), fully blocked 14-3-3ζ binding to ERK1/2 (Fig. 3d). To understand the molecular basis for the interaction between ERK2 docking groove and 14-3-3ζ D-site, we generated a three-dimensional model of ERK2 complexed to 14-3-3ζ by HADDOCK using the X-ray crystal structures of ERK2 and 14-3-3ζ protein^25,26^. As shown in Fig. 3e, side chains of MAPK docking groove in ERK2 about each other and adopt loop-like and α helix-like conformations that are exposed at the surface of ERK2. Main residues in D-site of 14-3-3ζ confront the elongated loop and α helix structures of MAPK docking groove to form an interface with ERK2. The ERK2-14-3-3ζ interaction is anchored by hydrophobic bonds for which residues T159, A314, Q315, and P311 of ERK2 (yellow) pack against residues F104, L100, L98, I93, N95, and T88 of 14-3-3ζ (green) or bound through electrostatic contacts for which the charged residues D318, D321, and K285 of ERK2 (yellow) pack against the charged residues K103 and D137 of 14-3-3ζ (green). These findings support the notion that MAPK docking grooves of ERK2 binds to D-site of 14-3-3ζ under hypoxic conditions.

Sequence analysis of 14-3-3ζ using Scansite online search programme (http://scansite.mit.edu/) validated that the N-terminus of 14-3-3ζ had a highly conserved MAPK phosphorylation site at the serine37 (S37) residue (Supplemental Fig. S3c). WB of the immunoprecipitated Flag with an anti-phospho-serine antibody revealed that deactivation of ERK1/2 with U0126, which by itself restores the 14-3-3ζ-YAP assembly (Fig. 2b), rather than inhibition of NF-κB with BAY, blocked the hypoxia-stimulated Ser phosphorylation of Flag-tagged wild-type 14-3-3ζ (Supplemental Fig. S4a). Likewise, depletion of ERK2 using siRNA abolished the hypoxia-stimulated 14-3-3ζ Ser phosphorylation almost as similarly as seen with U0126 (Fig. 3f). To directly elucidate whether ERK2 phosphorylates 14-3-3ζ at Ser37, we western-blotted the IPs of Flag-tagged wild-type 14-3-3ζ, mutant 14-3-3ζ S37A (in which S37 residue was mutated into a nonphosphorylatable alanine [A]) and S37E (in which S37 residue was mutated into a phosphorylation-mimetic glutamine [E]) in the presence or absence of rERK2 incubation with a developed rabbit polyclonal antibody that specifically recognizes S37 phosphorylation of 14-3-3ζ (14-3-3ζ p-S37) (Supplemental Fig. S4b, c). Figure 3g depicted that the mutant S37A is sufficient to mimicking U0126 or ERK2 siRNA for perturbing the ability of hypoxia to phosphorylate 14-3-3ζ because 14-3-3ζ S37A failed to undergo phosphorylation to the same extent as its wild-type counterpart did under hypoxia, and even so, ERK2 was able to phosphorylate the wild-type 14-3-3ζ but not the mutant 14-3-3ζ S37A (Fig. 3h). These results verify that ERK1/2 directly phosphorylates 14-3-3ζ at Ser37 in response to hypoxia.

### Phosphorylation at Ser37 disassembles 14-3-3ζ from YAP and promotes nuclear YAP localization during hypoxia

**Fig. 4:**
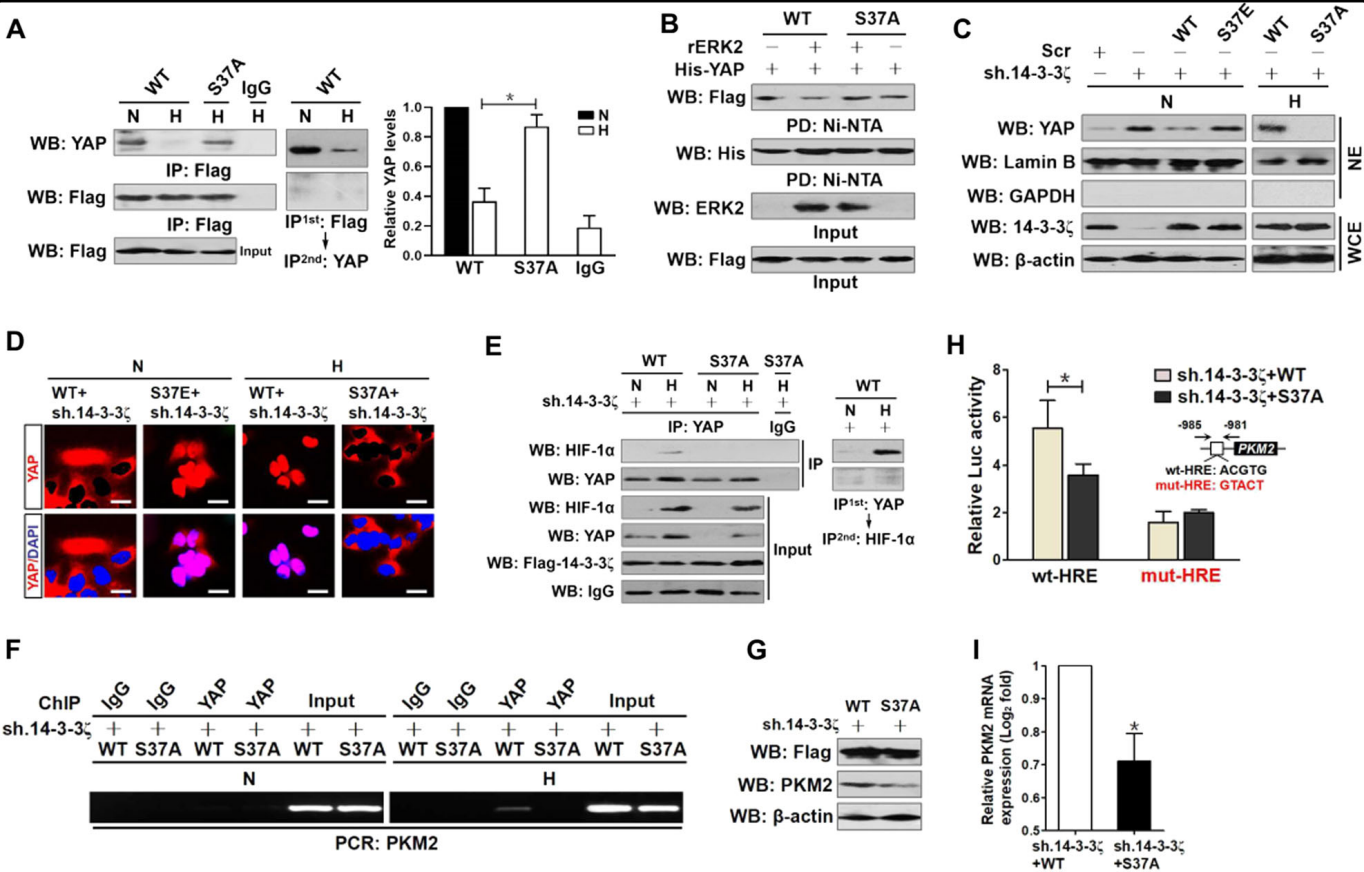
Phosphorylation at Ser37 disassembles 14-3-3ζ from YAP and promotes nuclear YAP localization during hypoxia, which facilitates PKM2 transcription via physical interaction with HIF-1α. **a** Coimmunoprecipitation assay evaluating the interaction of Flag-tagged wild-type 14-3-3ζ (WT) or mutant 14-3-3ζ S37A (S37A) with YAP in hypoxia-stimulated SW-1990 PDAC cells using an anti-YAP antibody. Data are expressed as mean ± s.d. of three independent experiments. *P < 0.05. Two-sided ANOVA with Bonferroni post hoc t test correction was used to calculate the *P* value. **b** In vitro protein binding assay for evaluation of 14-3-3ζ-YAP interaction with mixing purified His-tagged YAP immobilized on Ni^2+^-nitrilotriacetic acid (NTA)-sepharose beads and IPs of Flag-tagged wild-type 14-3-3ζ (WT), mutant 14-3-3ζ S37A (S37A) in the presence or absence of recombinant ERK2 protein (rERK2) followed by WB analyses with an anti-Flag antibody. PD, pull-down. **c** Subcellular fractionation analyses comparing nuclear YAP localization in 14-3-3ζ shRNA (sh.14-3-3ζ)-expressed SW-1990 PDAC cells with Flag-tagged wild-type 14-3-3ζ (WT), mutant 14-3-3ζ S37A (S37A) or S37E (S37E) reconstitution under normoxia and hypoxia. **d** Representative immunfluorescence images of nuclear YAP localization in 14-3-3ζ shRNA (sh.14-3-3ζ)-expressed SW-1990 PDAC cells with Flag-tagged wild-type 14-3-3ζ (WT), mutant 14-3-3ζ S37A (S37A) or S37E (S37E) reconstitution under normoxia and hypoxia. Scale bar = 25 μm. **e** Coimmunoprecipitation assay determining the interaction between nuclear YAP and HIF-1α in 14-3-3ζ shRNA (sh.14-3-3ζ)-expressed SW-1990 PDAC cells with Flag-tagged wild-type 14-3-3ζ (WT) or mutant 14-3-3ζ S37A (S37A) reconstitution under hypoxia. **f** ChIP analysis for YAP binding to PKM2 gene HRE in 14-3-3ζ shRNA (sh.14-3-3ζ)-expressed SW-1990 PDAC cells with hypoxia stimulation in the presence or absence of Flag-tagged wild-type 14-3-3ζ (WT) or mutant 14-3-3ζ S37A (S37A) reconstitution using the indicated antibodies. **g** Western-blotting examining abundance of Flag and PKM2 protein expression in 14-3-3ζ shRNA (sh.14-3-3ζ)-expressed SW-1990 PDAC cells with hypoxia stimulation in the presence or absence of Flag-tagged wild-type 14-3-3ζ (WT) or mutant 14-3-3ζ S37A (S37A) reconstitution. **h** Luciferase-reporter PKM2 HRE activity analysis of 14-3-3ζ shRNA (sh.14-3-3ζ)-expressed SW-1990 PDAC cells with hypoxia stimulation in the presence or absence of Flag-tagged wild-type 14-3-3ζ (WT) or mutant 14-3-3ζ S37A (S37A) reconstitution. Data are expressed as mean ± s.d. *P < 0.05. Two-sided ANOVA with Bonferroni post hoc t test correction was used to calculate the *P* value. **i** RT-qPCR analyses of PKM2 gene expression in 14-3-3ζ shRNA (sh.14-3-3ζ)-expressed SW-1990 PDAC cells with hypoxia stimulation in the presence or absence of Flag-tagged wild-type 14-3-3ζ (WT) or mutant 14-3-3ζ S37A (S37A) reconstitution. Experiments were performed five times, each with quantitative RT-PCR in technical duplicate and real-time values were normalized to glyceraldehyde 3-phosphate dehydrogenase (GAPDH). Data are expressed as mean ± s.d. *P < 0.05. Two-sided Student’s *t* test was used to calculate the *P* value

Next, we compared YAP abundance in IPs of wild-type 14-3-3ζ and mutant 14-3-3ζ S37A to decipher whether 14-3-3ζ Ser37 phosphorylation disrupts 14-3-3ζ-YAP assembly. Introduction of cells with mutant S37A made them refractory to the hypoxia-stimulated disassembly of 14-3-3ζ from YAP (Fig. 4a). As estimated by in vitro protein binding assay and shown in Fig. 4b, wild-type 14-3-3ζ lost its ability to interact with YAP in the presence of ERK2, whereas the phosphorylation-defective 14-3-3ζ S37A mutant persistently binds to YAP regardless of ERK2 incubation. In an attempt to test the role of 14-3-3ζ Ser37 phosphorylation in YAP nuclear translocation, we depleted e14-3-3ζ using short hairpin RNA (shRNA) targeting 14-3-3ζ (sh.14-3-3ζ) (Supplemental Fig. S4d) and reconstituted these cells with wild-type 14-3-3ζ, mutant 14-3-3ζ S37E or S37A in the presence or absence of hypoxia, respectively. Ablation of e14-3-3ζ by shRNA under normoxic conditions, as determined by subcellular fractionation (Fig. 4c) and IF analyses (Fig. 4d), greatly augmented nuclear YAP accumulation, the phenotype that could be abrogated by reconstituted expression of wild-type 14-3-3ζ instead of 14-3-3ζ S37E mutant. Although hypoxia stimulated YAP nuclear translocation in the e14-3-3ζ-depleted cells reconstituted with wild-type 14-3-3ζ, it was unable to do so in the depleted cells with mutant 14-3-3ζ S37A reconstitution. Hence, 14-3-3ζ Ser37 phosphorylation disassembles 14-3-3ζ from YAP and promotes YAP to localize in nucleus during hypoxia.

### YAP facilitates PKM2 transcription via physical interaction with HIF-1α inan 14-3-3ζ Ser37 phosphorylation-dependent manner under hypoxia

Based on the aformetioned data showing that YAP redistributes to nucleus upon hypoxia and the fact that hypoxia stabilizes nuclear HIF-1α protein^27^, we investigated the intrinsic association between YAP and HIF-1α. WB of the immunoprecipitated YAP from nuclear fractions in hypoxia-stimulated S-1 cells with an anti-HIF-1α antibody demonstrated that YAP physically interacts with HIF-1α at endogenous levels under hypoxia per se, while this interaction was absent after depletion of ERK2 with siRNA (Supplemental Fig. S5a). In agreement with these findings, reconstituted expression of the mutant 14-3-3ζ S37A, but not of its wild-type counterpart, in the e14-3-3ζ-depleted cells blocked the YAP-HIF-1α interaction stimulated by hypoxia (Fig. 4e). Unexpectedly, the interaction between YAP and HIF-1α in e14-3-3ζ-depleted cells with mutant 14-3-3ζ S37E reconstitution, which in the case of normoxia, was absent and did not differ from that in the depleted cells with or without wild-type 14-3-3ζ reconstitution, presumably attributing to the HIF-1α deficiency under normoxic conditions (Supplemental Fig. S5b). These results proposed that the ERK2-dependent 14-3-3ζ Ser37 phosphorylation allows nuclear YAP-HIF-1α interaction upon hypoxia.

The hypoxia-inducible interaction between YAP and HIF-1α mediated by 14-3-3ζ Ser37 phosphorylation raises the possibility that YAP might facilitate HIF-1α transcriptional activation in a Ser37 phosphorylation-dependent manner. To interrogate whether YAP is a transcriptional coactivator with HIF-1α, we explored the role of YAP in HIF-1α recruitment at HRE of PKM2 gene (a downstream target of HIF-1α engaged in catalyzing glycolytic reaction) through chromatin immunoprecipitation (ChIP) assay with antibodies against HIF-1α, HIF-2α, or nonrelated IgG. Relative to IgG negative control, hypoxia augmented both HIF-1α and HIF-2α occupancy in the HRE region of PKM2, but only HIF-1α occupancy was prominently alleviated when YAP had been silenced with shRNA (Supplemental Fig. S5c), suggesting that the YAP-HIF-1α interaction might be instrumental and unique for PKM2 transcription. To gain insight into whether YAP facilitates HIF-1α‘s transcriptional activation at least partially in an 14-3-3ζ Ser37 phosphorylation-dependent manner, we carried out ChIP assay on the e14-3-3ζ-depleted cells reconstituted with wild-type 14-3-3ζ or mutant 14-3-3ζ S37A. In echoing the observation that phosphorylation of 14-3-3ζ at Ser37 promotes YAP nuclear localization, the mutant 14-3-3ζ S37A, which has a much-impaired ability to allow YAP-HIF-1α interaction, markedly reduced the hypoxia-stimulated recruitment of YAP at PKM2 HRE compared with wild-type 14-3-3ζ (Fig. 4f). The e14-3-3ζ-depleted cells with wild-type 14-3-3ζ or mutant 14-3-3ζ S37A reconstitution had comparable levels of Flag epitope expression (Fig. 4g), which may not account for the reduction in YAP and HIF-1α ChIP resulting from S37A mutant. Additional evidence that HIF-1α transactivates PKM2 in conjunction with YAP was delineated by the HIF-1α siRNA-transfected cells, whose YAP binding at PKM2 HRE was abolished upon hypoxia stimuli in comparison with the parental cells transfected with control siRNA (Supplemental Fig. S5d).

To address whether 14-3-3ζ Ser37 phosphorylation influences transcriptional activity of PKM2 in response to hypoxia, we enrolled a dual-luciferase reporter assay to determine the promoter activities of PKM2 gene in cells expressed pGL3-PKM2-Luc containing PKM2 promoter with either wild-type (wt) or mutant (mut) HRE sequence (from −985 to −981 nucleotides). Exogenous expression of mutant 14-3-3ζ S37A, but not wild-type 14-3-3ζ, in the e14-3-3ζ-depleted cells during hypoxia attenuated wt HRE activities by 1.5-fold without significant alteration of mut HRE activities (*P* < 0.05; Fig. 4h). Notably and in accordance with the data confirmed by dual-luciferase reporter assay, real-time quantitative reverse transcriptase-polymerase chain reaction (RT-qPCR) analysis using primers specific for PKM2 mRNA and WB analysis detected lower levels of PKM2 mRNA and protein in e14-3-3ζ-depleted cells harboring mutant 14-3-3ζ S37A than those in depleted cells harboring wild-type 14-3-3ζ under hypoxia (*P* < 0.05; Fig. 4i, g). As well, PKM2 mRNA and protein expression in the YAP shRNA-transfected cells was downregulated in contrast to those in cells transfecting with scrambled shRNA (Scr) in the context of hypoxia (*P* < 0.05; Supplemental Fig. S5e, f), implying that the hypoxia-dependent 14-3-3ζ Ser37 phosphorylation confers YAP-HIF-1α interaction and their recruitment at HRE of PKM2, for which PKM2 transcription is required.

### 14-3-3ζ Ser37 phosphorylation contributes to glycolysis and tumorigenesis of PDAC cells

**Fig. 5:**
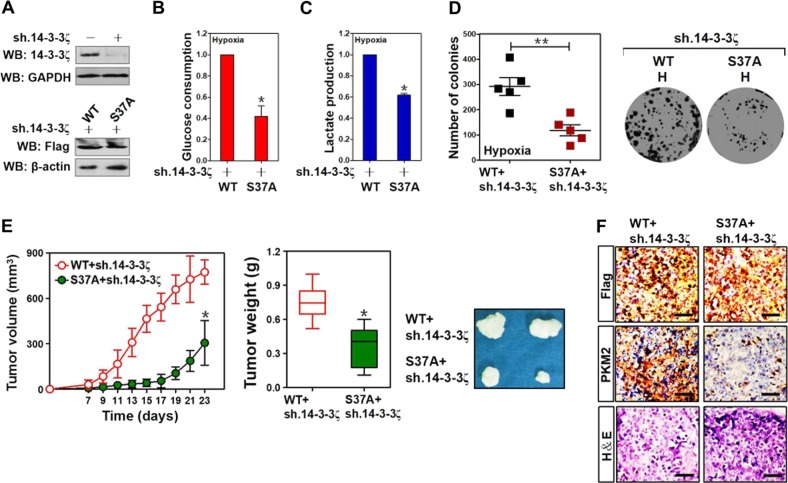
14-3-3ζ Ser37 phosphorylation contributes to glycolysis and tumorigenesis of PDAC cells. **a** *Top panel*: Western blotting assessing levels of 14-3-3ζ in SW-1990 PDAC cells with or without 14-3-3ζ shRNA (sh.14-3-3ζ) transfection. *Bottom panel*: Western blotting examining the expression levels of Flag in 14-3-3ζ shRNA (sh.14-3-3ζ)-expressed SW-1990 PDAC cells with Flag-tagged wild-type 14-3-3ζ (WT) or mutant 14-3-3ζ Ser37A (S37A) reconstitution. **b**, **c** Glucose consumption (**b**) or lactate production (**c**) of 14-3-3ζ shRNA (sh.14-3-3ζ)-expressed SW-1990 PDAC cells with hypoxia stimulation in the presence or absence of Flag-tagged wild-type 14-3-3ζ (WT) or mutant 14-3-3ζ S37A (S37A) reconstitution. Data are expressed as mean ± s.d. of at least three experiments. *P < 0.05. Two-sided Student’s t test was used to calculate the *P* value. **d** Colony formation assays of 14-3-3ζ shRNA (sh.14-3-3ζ)-expressed SW-1990 PDAC cells with hypoxia stimulation in the presence or absence of Flag-tagged wild-type 14-3-3ζ (WT) or mutant 14-3-3ζ S37A (S37A) reconstitution. **P < 0.01. Two-sided Student’s t test was used to calculate the *P* value. **e** Tumor volume (left panel), weight (middle panel) and representative images (right panel) of xenografts excised from the tumor-bearing mice. Indicated SW-1990 PDAC cells (1 × 10^7^) were subcutaneously inoculated into the right flank of nude mice. The mice were sacrificed and the tumors were excised and measured on day 23. Data are presented as mean ± s.d. *P < 0.05 versus WT + sh.14-3-3ζ. Two-sided Student’s *t* test was used to calculate the *P* value. **f** Representative IHC staining images for PKM2 and H&E staining in the indicated SW-1990 PDAC tumors from mice at day 23 after inoculation. Scale bar = 100 μm

PKM2 is responsible for increasing glucose uptake and converting pyruvate into lactate, a phenomenon so-called glycolysis^28^. Along with the contributing role of 14-3-3ζ Ser37 phosphorylation in PKM2 transcription, the e14-3-3ζ-depleted cells with mutant 14-3-3ζ S37A reconstitution had lower magnitude of glucose uptake and lactate production than the depleted cells reconstituted with wild-type 14-3-3ζ under hypoxia (*P* < 0.05; Fig. 5a-c and Supplemental Fig. S5g), leading us to the proposal that 14-3-3ζ Ser37 phosphorylation elicits transcriptional upregulation of PKM2, thus promoting the hypoxia-dependent glycolysis in PDAC cells.

Take into account that 14-3-3ζ Ser37 phosphorylation promotes glycolysis during hypoxia, we sought to understand its significance in cell proliferation of PDAC. Upon hypoxia, reconstitution of the mutant 14-3-3ζ S37A more pronouncedly suppressed the number and size of surviving colonies in the e14-3-3ζ-depleted cells than did wild-type 14-3-3ζ (*P* < 0.01; Fig. 5d). We also examined the role of 14-3-3ζ Ser37 phosphorylation in vivo tumorigenicity by subcutaneously inoculating the e14-3-3ζ-depleted cells with wild-type 14-3-3ζ or mutant S37A reconstitution into BALB/c nude mice. Phosphorylation of 14-3-3ζ at Ser37 empowered a prominent acceleration for PDAC development in mice as xenografts burdened by the e14-3-3ζ-depleted cells with mutant 14-3-3ζ S37A reconstitution displayed statistically significant smaller volume (*P* < 0.05) and lighter weight (*P* < 0.05) at 23 days after inoculation than those burdened by the depleted cells with wild-type 14-3-3ζ reconstitution (Fig. 5e). Further histopathological analyses confirmed that the e14-3-3ζ-depleted xenografts with mutant S37A reconstitution presented lower PKM2 staining than the depleted xenografts reconstituting with wild-type 14-3-3ζ (Fig. 5f). These data implicate that 14-3-3ζ Ser37 phosphorylation-mediated PKM2 upregulation promotes glycolysis and tumorigenesis of PDAC cells under in vitro hypoxic conditions and in vivo tumorigenic microenvironment.

### 14-3-3ζ Ser37 phosphorylation positively correlates with p-ERK1/2 activity and HIF-1α expression in clinical samples from patients with PDAC and predicts unfavorable prognosis

**Fig. 6:**
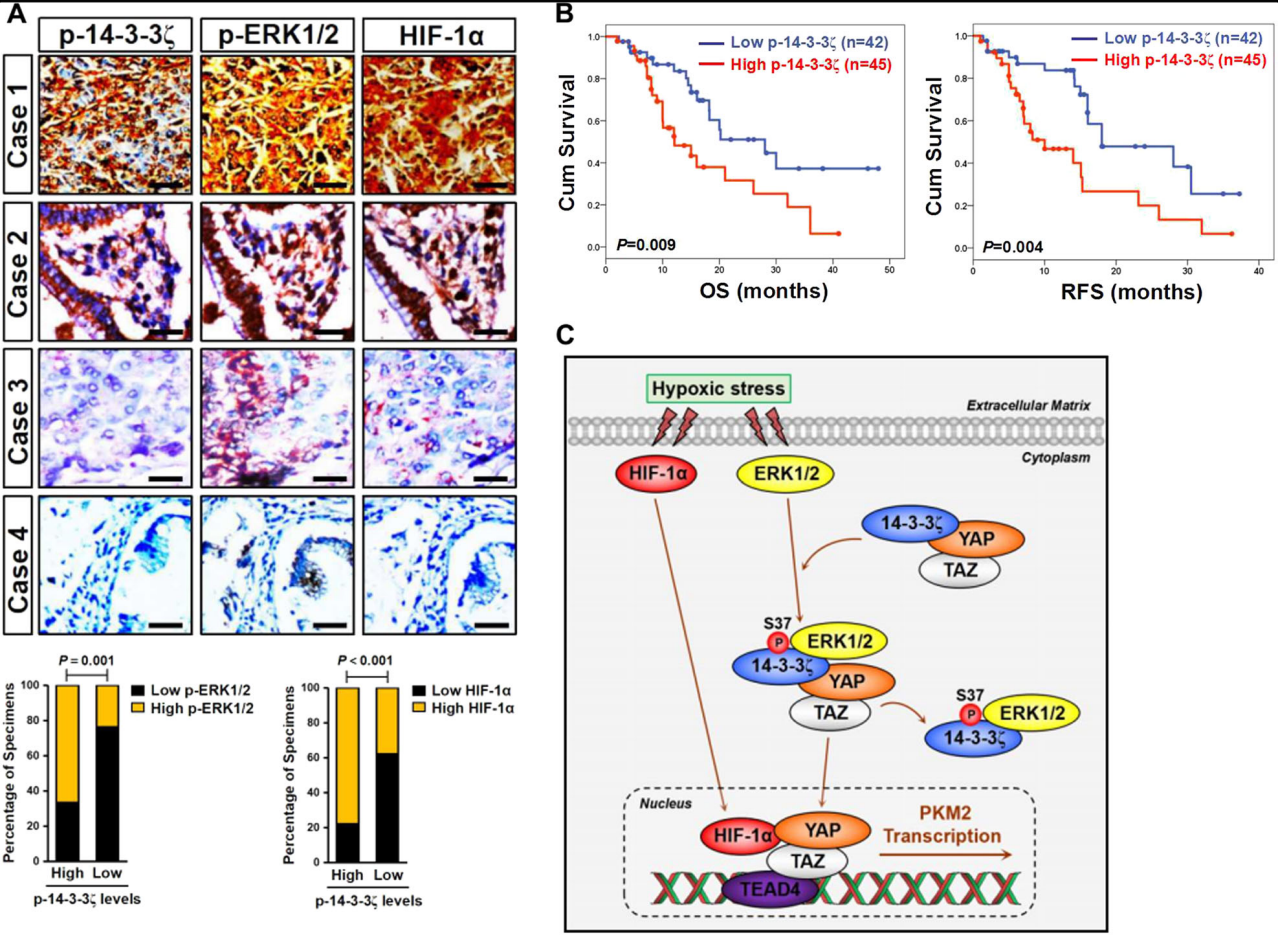
14-3-3ζ Ser37 phosphorylation positively correlates with p-ERK1/2 activity and HIF-1α expression in clinical samples from patients with PDAC, and it predicts unfavorable prognosis. **a** Representative cases stained by immunohistochemistry (top panel) and bar graph (bottom panel) showing the expression of 14-3-3ζ p-S37 in 87 primary human PDAC specimens is positively correlated with the levels of p-ERK1/2 and HIF-1α. Scale bar = 50 μm. The *P* value shown was calculated by Spearman order correlations. **b** Kaplan-Meier curves comparing the overall survival (OS) and recurrence free survival (RFS) in 87 PDAC patients with low and high expression of 14-3-3ζ p-S37. Log-rank test was used to calculate the *P* value. **c** Proposed schematic illustrating a pivotal role for the ERK2-induced 14-3-3ζ Ser37 phosphorylation in promoting nuclear YAP localization and driving PKM2 transcription via physical YAP-HIF-1α interaction in response to hypoxic stress

Lastly, we wondered whether our above-stated findings have clinical relevance. For this purpose, we carried out immunohistochemistry (IHC) on 87 sections of PDAC specimens from clinical patients (Supplemental Table S1) to determine the relationship between 14-3-3ζ Ser37 phosphorylation, p-ERK1/2 activity, and HIF-1α expression using antibodies against 14-3-3ζ p-S37, HIF-1α, and p-ERK1/2, respectively. To ensure the 14-3-3ζ Ser37 phosphorylation also exists in human PDAC tissues, we tested the applicability of the anti-14-3-3ζ p-S37 antibody for IHC. We found that the staining of p-14-3-3ζ S37 could be blocked by phosphorylated 14-3-3ζ peptide used for developing the antibody but not by the control nonphosphorylated peptide (Supplemental Fig. S5h). Among these specimens, 45 out of 87 cases (51.7%) were positive, and 42 out of 87 cases (48.3%) were negative for 14-3-3ζ p-S37. Of note, 33.3% (15 cases) of samples with high 14-3-3ζ p-S37 expression exhibited low levels of p-ERK1/2, whereas 30.9% (13 cases) of samples with low 14-3-3ζ p-S37 expression had high expression of p-ERK1/2, respectively (*r* = 0.357, *P* = 0.001; Fig. 6a). Meanwhile, positive 14-3-3ζ p-S37 was detected in 77.8% specimens with strong expression of HIF-1α and in 22.2% specimens with weak expression of HIF-1α, while negative 14-3-3ζ p-S37 was detected in 38.0% specimens with strong expression of HIF-1α and 62.0% specimens with weak expression of HIF-1α (*r* = 0.403, *P* < 0.001; Fig. 6a). In addition, there was a strong correlation between the levels of 14-3-3ζ p-S37 and nuclear YAP localization in 56 samples among the 87 PDAC tissues (*r* = 0.430, *P* = 0.001), where 59.4% samples (19/32) with nuclear YAP localization displayed high 14-3-3ζ p-S37 expression levels, while only 16.7% samples (4/24) with nuclear YAP localization displayed low 14-3-3ζ p-S37 expression levels (Supplemental Fig. S5i). Despite no relationship between 14-3-3ζ p-S37 and gender (*P* = 0.313), tumor location (*P* = 0.103), advanced clinical stage (*P* = 0.200), as well as adjuvant chemotherapy (*P* = 0.104) was observed in these specimens, phosphorylation of 14-3-3ζ significantly correlated with older patients (*P* = 0.015), tumor size (*P* < 0.001) and lymph node metastasis (*P* = 0.031), respectively (Supplemental Table S2). Follow-up analysis of survival durations in the 87 PDAC specimens showed that patients whose tumors displayed high 14-3-3ζ Ser37 phosphorylation had a statistically significant shorter overall survival (OS) and recurrence free survival (RFS) than those with low 14-3-3ζ Ser37 phosphorylation in their tumors (Fig. 6b), quantitatively corresponding to the median times 12.07 ± 3.35 months versus 28.02 ± 7.69 months for OS (*P* = 0.009; Log-rank test) and 10.00 ± 3.44 months versus 18.00 ± 6.23 months for RFS (*P* = 0.004; Log-rank test), respectively. These results suggest that 14-3-3ζ Ser37 phosphorylation positively correlates with p-ERK1/2 activity and HIF-1α expression in clinical samples from patients with PDAC and predicts unfavorable prognosis.

## Discussion

The progression from a non-cancer cell to a cancer cell is a complicated process in which at least six hallmarks changes direct the malignant transformation. How distinct defects synergistically generate a neoplastic lesion remains a long-standing unanswered question in cancer biology. A better understanding of molecular signal transduction mechanisms might lay framework for developing innovative therapies. PDAC remains a lethal malignancy yet advances in recent studies concerning its biological characterization^29,30^. Given both hypoxic microenvironment and YAP transcriptional activation are intimately wired to malignant phenotypes, we postulate that nuclear YAP serves as a “stress sensor” that cooperates with HIF-1α to drive hypoxia-responsive gene transcription and govern PDAC progression under hypoxic microenvironment.

In the current study, using biochemical and genetic approaches, we demonstrate what we believe the first that the nuclear YAP translocation mediated by ERK2-initiated 14-3-3ζ Ser37 phosphorylation exerts a contributing role on the HIF-1α-dependent glycolysis and tumorigenicity of PDAC cells based on the following evidences: (1) Hypoxia stimuli disassembles 14-3-3ζ from YAP and thereby triggers nuclear translocation of YAP via activating ERK2; (2) The MAPK docking grooves of ERK2 directly interact with the D-site Leu98/100 in 14-3-3ζ and subsequently phosphorylates 14-3-3ζ at Ser37, as reflected by the result that 14-3-3ζ D-site mutant loses its ability to bind to ERK1/2 and the data that 14-3-3ζ fails to undergo the ERK2-induced phosphorylation when its Ser37 is mutated into a phospho-defective alanine (A); (3) Genetic ablation of YAP, as equivalently as reconstituted expression of 14-3-3ζ S37A, antagonizes the HIF-1α-dependent PKM2 transcription under hypoxia; (4) The e14-3-3ζ-depleted cells with 14-3-3ζ S37A reconstitution exhibit lower glucose uptake, lactate production, as well as delayed tumor growth than their counterparts, presumably owing to transcriptional downregulation of PKM2; (5) 14-3-3ζ Ser37 phosphorylation not only positively correlates with p-ERK1/2 activity and HIF-1α expression in clinical PDAC samples but also predicts poor prognosis for patients with PDAC. To our knowledge, these data together provide a rationale that the ERK2-mediated and 14-3-3ζ Ser37 phosphorylation-mediated YAP nuclear translocation sustains glycolysis and tumorigenesis of PDAC via HIF-1α-dependent PKM2 transcription during hypoxia. Our future work will aim at unveiling whether the other molecules are essential for the hypoxia-initiated disassembly of 14-3-3ζ from YAP and ascertaining the exact mechanisms of how nuclear YAP acts as a coactivator for HIF-1α to reprogram glucose metabolism in PDAC cells.

Although the molecular mechanism underlying YAP nuclear translocation is still not comprehensively studied, the concept that 14-3-3ζ binding to YAP forms a heterodimer and sequesters YAP in cytoplasm has been documented in a large number of literature. As a member of the evolutionally conserved 14-3-3 family, 14-3-3ζ binds to RxxpT/pSxP motif within substrate proteins so as to recruit numerous TFs engaging in cancer development^31^. Meanwhile, the finding that 14-3-3ζ controls nuclear transport of substrate proteins has been implicated in many biological and pathological processes. For instance, 14-3-3ζ has been reported to control nuclear translocation of βII protein kinase C (PKC) anchoring protein RACK1 by interactions with the amino acid residues that span the propeller blades WD2-3 and WD4-5 of RACK1^32^. It was also demonstrated to regulate nuclear inclusion of protein phosphatase 1α (PP1α) via a mechanism independent of PP1α phosphorylation^33^. Our study identifies a mechanism whereby 14-3-3ζ promotes nuclear YAP localization through ERK2-dependent autophosphorylation, and the explicit details for this process are warranted to be defined.

Besides the serine/threonine phosphorylation event in the amino acid sequence that is critical for 14-3-3 function^34^, it has been reported that 14-3-3 can be phosphorylated at distinct residues by several protein kinases, such as calcium-dependent protein kinase (CPK), c-Jun N-terminal kinase (JNK), and casein kinase (CK)^35,36^. In the present study, detection of wild-type 14-3-3ζ, but not that of unphosphorylatable 14-3-3ζ Ser37A mutant by antibody against phospho-Ser37-14-3-3ζ peptide validated its specificity, which is consistent with the IHC staining in human PDAC tissues. Our results show hypoxia stimulates 14-3-3ζ Ser37 phosphorylation via activating ERK2. Intriguingly, this phenotype is not coupled with ROS production and NF-κB activation, the two signaling components responsible for balancing survival/death transition as a result of hypoxic stress. This interpretation is coincide with the proposed model for ERK2-14-3-3ζ proteins docking, in which ERK2-14-3-3ζ interaction is disrupted when the D-site Leucine98/100 residues of 14-3-3ζ are replaced by alanines. Herein, the possibility that phosphorylation of 14-3-3ζ by ERK2 releases YAP is emerged from the observation that the phospho-defective S37A compromises, while the phospho-mimetic S37E potentiates YAP nuclear localization in e14-3-3ζ-depleted cells. Whether additional residues of 14-3-3ζ, particularly tyrosine as mentioned in other researches^37^, are engaged in YAP subcellular redistribution under hypoxia still needs further investigation.

Intratumoral HIF-1α accumulation is commonly found in solid tumors, which leads to target genes transcription with or without recruitment of TFs at their HREs. On the other hand, nuclear YAP localization in response to hypoxic stress likely enables YAP to be in complex with different transcriptional coregulators and to induce different sets of gene expression through TEAD4. For instance, YAP binds to and stabilizes HIF-1α in nucleus during hypoxic stress, thus accelerating hepatic tumorigenesis^38^, while the interaction of YAP/TAZ with TEAD4 releases VGLL4 and augments the TEAD4-dependent gene transcription to sustain tissue growth and repress cell apoptosis^39^. The nuclear localization of YAP evoked by 14-3-3ζ Ser37 phosphorylation seems to be a pivotal step for HIF-1α to transactivate PKM2. Functional interdependence between HIF-1α and PKM2 has been recently reported in mouse embryonic fibroblasts (MEFs), where the prolyl hydroxylase 3 (PHD3)-mediated prolyl hydroxylation of PKM2 induces formation of a macromolecular complex encompassing HIF-1α, p300, as well as PKM2 and thereby transactivates PKM2. In this regard, it would be of interest to note that in the current study, YAP and HIF-1α colocalization is detected in nucleus and they coordinately augment transcription of PKM2 gene which is characterized to increase glucose uptake and lactate production in PDAC cells. Interestingly, YAP shares many conserved domains, such as the WW domain, coiled-coil region, and PDZ-binding motif with WWTR1, which acts as a powerful coactivator for HIF-1α^40^. Moreover, loss of HIF-2α fails to influence the YAP-dependent PKM2 transcription in the context of hypoxia (data not shown). One speculation is HIF-1α serves as a partner in such coactivating event which may not be achieved by other molecules. As TEAD4 mediates transcriptional activity of YAP^41^, it is still not known whether hypoxia triggers formation of three distinct complexes or a ternary complex consisting of YAP-TEAD4-HIF-1α with all their properties. The physical interaction and recruitment of YAP at HRE of PKM2 promoter with HIF-1α presented in our study illustrate a possible mechanism whereby YAP cooperates with HIF-1α to reprogram glucose metabolism and bypasses tricarboxylic acid (TCA) cycle, thus extending the biological role of YAP, beyond its previously appreciated behaviors in tissue regeneration and organ differentiation^42,43^, to a glycolytic role during PDAC progression.

There are some limitations in our work. First, despite our data clearly demonstrate that 14-3-3ζ is instrumental and sufficient for blocking the nuclear import of YAP under hypoxic conditions, this may not be the only mechanism as YAP is known to be anchored in cytoplasm by other 14-3-3 isoforms^44^. It is worth unearthing the impact of other 14-3-3 isoforms on the subcellular YAP redistribution during hypoxia. Second, protein kinase C delta (PKCδ) directly interacts with and phosphorylates 14-3-3ζ at Ser58^45^, so the possibility that PKCδ participates in the hypoxia-initiated YAP nuclear inclusion could not be ruled out. It is conceivable that PKC might play a principal role in the coactivating function of YAP-HIF-1α and that other members of MAPK family may regulate 14-3-3ζ phosphorylation in different cell types.

In summary, our work shed light on the notion that the ERK2-mediated 14-3-3ζ Ser37 phosphorylation augments HIF-1α-dependent PKM2 transcription and glycolysis via YAP in PDAC cells under hypoxic microenvironment (Fig. 6c). The metabolic shift critical for tumorigenesis by 14-3-3ζ Ser37 phosphorylation refers to exploitation of its unestablished function. If so, 14-3-3ζ phosphorylation might become an attractive target for therapeutic intervention in PDAC. Our studies also underscore the significance of hypoxia-responsive ERK2-14-3-3ζ macrocomplex in guiding the metabolism-based cancer therapy.

## Materials and methods

### Cell culture and transfection

The culture protocols for human SW-1990 PDAC and Huh-7 HCC cells were described in previous publications^46,47^. Briefly, SW-1990 and Huh-7 cells were cultured in high-glucose DMEM (Gibco, Carlsbad, USA) containing 10% FBS, 100 mg/mL penicillin and streptomycin and cultured at 37 °C in 5% CO_2_ and 95% air. A498 cells were grown in 90% RPMI1640 (Gibco, USA) supplemented with 10% fetal bovine serum (FBS), 100 U/mL penicillin, and 100 mg/mL streptomycin at 37 °C and 5% CO_2_ in a humidified atmosphere. Physical hypoxia were achieved with an O_2_/CO_2_ incubator (Billups-Rothenberg) containing a gas mixture composed of 1% O_2_, 5% CO_2_, and 94% N_2_. For chemical hypoxia induction, the indicated cells were exposed to different concentrations of CoCl_2_ for 12 h and then harvested for further analyses. Transient and stable transfection were performed according to previous reports^46^. For transient transfection, cells seeded in six-well plate were transfected with the indicated plasmids or siRNAs using Lipofectamine 2000 reagent in OPTI-MEM medium (Invitrogen). The medium was then replaced with fresh medium after 6 h and the cells were harvested for further analyses 48 h later. The stable cell clones were selected with G418 (600 μg/mL) or puromycin (1 μg/mL) for 2 weeks.

### Reagents and antibodies

U0126 (purity > 98%, #9903) was purchased from Cell Signaling Technology (CST, USA). BAY 11-7085 (purity > 98%, cas14795) was from Cayman Chemical (Ann Arbor, MI) and recombinant ERK2 kinase (cat# 14-550 M) was ordered from Millipore (Billerica, MA). Lipofectamine 2000 was from Invitrogen (Carlsbad, CA, USA) and 4’,6-Dimidino-2-phenylindole dihydrochloride (DAPI) was purchased from KeyGene Biotech (Nanjing, China). The antibodies against YAP (monoclonal, #14074), phospho-ERK1/2 (monoclonal, #9106), phospho-IκBα (monoclonal, #2859), and Lamin B (monoclonal, #13435) were purchased from Cell Signaling Technology (Danvers, MA). The antibodies against 14-3-3ζ (monoclonal, sc-293415), ERK2 (monoclonal, sc-136288) and WWTR1 (monoclonal, sc-293183) were from Santa Cruz Biotechnology (Santa Cruz, CA). The antibodies against HIF-1α (polyclonal, ab2185), phospho-serine (polyclonal, ab9332), and PKM2 (monoclonal, ab150377) were from Abcam (Cambridge, UK). The phospho-14-3-3ζ Ser37 antibody was produced using the synthetic phosphorylated peptides VTEQGAELpSNEERNLL (Peptide 2.0 Inc, Chantilly, VA) as antigen and purified on a phosphopeptide column (EZBiolab Inc, Carmel, IN). The antibodies against GAPDH and β-actin were obtained from Biosynthesis (Beijing, China).

### Plasmids and reporter gene construct

The vector expressing Myc-TEAD4 (pReceiver-M43) and short hairpin RNA (shRNA) targeting YAP (psi-nH1) were ordered from Genecopoeia (Rockville, MD). PCR-amplified human14-3-3ζ was subcloned into pFlag-CMV^TM^-2 vector (Sigma, St Louis, MO) for transient transfection and c-Flag pcDNA 3 vector (Addgene, Cambridge, MA) for stable transfection. 14-3-3ζ L98/100 A, S37A, and S37E were generated using a QuickChange^®^ Site-Directed Mutagenesis Kit (Agilent Technologies, Santa Clara, CA). The PKM2 promoter luciferase reporter gene was constructed by cloning the wild-type or mutant HRE region of PKM2 into SacI/XmaI sites of pGL3 luciferase reporter plasmid (Promega, Madison, WI) with the following primers: wt-HRE: 5′-CGAGCTCGAGTTGAGACCATCCTGGCCAA-3′(forward) and 5′-CCCGGGGAAGACGGGGTTTCGCCACGT-3′ (reverse). mut-HRE: 5′-CGAGCTCGGTTGAGACCATCCTGGCCAGT-3′ (forward) and: 5′-CCCG

GGGA GCCAAGACGGGGTTCTCAAC-3′(reverse). Small interfering RNA (siRNA) targeting 14-3-3ζ and HIF-1α were purchased from GenePharma (Shanghai, China). ERK2 siRNA (#6578) was obtained from Cell Signaling Technology.

### Co-IP and WB

Co-IP assays and WB analyses were carried out according to the detailed procedures as described with some modifications in previous studies^47^. For co-IP, cells washed twice with cold PBS were solubilized on ice in a radioimmunoprecipitation assay (RIPA) buffer (Cwbiotech, Beijing, China) containing 50 mM Tris [PH7.4], 150 mM NaCl, 1% NP-40, 0.25% sodium deoxycholate, and protease inhibitors. Following brief sonication, the lysates were centrifuged with 15,000 *×* *g* speed for 15 min at 4 °C and the supernatants were subsequently incubated with antibody against the indicated primary antibodies together with protein A/G-sepharose beads (Cwbiotech, Beijing, China) overnight at 4 °C. After immunoprecipitation, the beads were washed three times with washing buffer (50 mM Tris-HCl, pH 7.6; 300 mM NaCl; 1 mM EDTA; 0.5% NP-40; 10% glycerol), then the precipitates were boiled for 10 min in sample buffer and analyzed by WB. The primary antibodies were replaced with nonimmune normal rabbit IgG in control samples. For WB, cells were pelleted by centrifugation and rinsed with PBS to prepare the cytoplasmic proteins. Pellets were then resuspended in 500 μl ice-cold RIPA buffer containing protease inhibitors and phosphatase inhibitors (KeyGen BioTech, Nanjing, China) by pipetting up and down about 10 times. Following incubation on ice for 10 min, the lysates were centrifuged at 15,000 × *g*. The supernatants were then transferred to another fresh tubes and referred to as cytoplasmic extracts. To isolate nuclear proteins, the pellets were vigorously resuspended in nuclear protein extraction buffer with inhibitors (Vazyme BioTech, Nanjing, China) and centrifuged for 10 min at 15,000×*g* at 4 °C. Proteins were fractionated on 10% sodium dodecyl sulfate-polyacrylimide gel electrophoresis (SDS-PAGE) and transferred to the Immobilon™ PVDF Transfer Membranes (Millipore Corporation, Billerica, MA). After blocked in 5% bovine serum albumin (BSA), the membrane was incubated with the primary antibodies overnight and followed by HRP-linked secondary antibodies incubation for 1 h. The bands were then visualized by western chemiluminscent HRP substrate kit (PPLYGEN, Beijing, China).

### In vitro protein kinase assay

Cells were transfected with constructs expressing Flag-tagged wild-type 14-3-3ζ or 14-3-3ζ Ser37A mutant. Forty-eight hours later, wild-type or mutant 14-3-3ζ was purified and then incubated with 1 µg of recombinant GST-ERK2 fusion protein in the presence of 200 µM cold ATP for 30 min. The reaction was terminated by the addition of SDS-containing lysis buffer and the reaction products were resolved by SDS-PAGE and detected by WB.

### Immunofluorescence

IF was measured using standard methods as reported previously^46^. In brief, the indicated cells were cultured in six-well plates and fixed with 4% formaldehyde in PBS for 10 min at 37 °C. After permeabilizing with 1% Triton X-100 in PBS for 10 min, cells were blocked with 5% BSA in PBS and incubated with primary antibody overnight. Cells were then incubated with Alexa Fluor^®^ 594 conjugate secondary antibody (1:1000, Cell Signaling Technology) at 37 °C for 1 h. Nuclei were stained with 5 µg/mL DAPI for 15 min and the staining was viewed with an IX71 fluorescent microscope (Olympus, Japan).

### GST-pulldown assay

Immunoprecipitates from PDAC cells expressing Flag-tagged wild-type 14-3-3ζ were incubated with 100 ng of purified GST or GST fusion protein in a kinase buffer containing 50 mM ATP, 150 mM NaCl, 1 mM dithiothreitol, 0.5 mM EDTA, 0.1 mM phenylmethylsulfonyl fluoride, 100 μM sodium vanadate, and 1 mM sodium fluoride for 30 min at 30 °C. The GST-bound proteins were eluted by glutathione agarose beads and then subjected to SDS-PAGE and blotted with the indicated antibodies.

### Chromatin immunoprecipitation

ChIP assays were determined as described previously^47^. Briefly, cells in each group were treated with 1% formaldehyde and disuccimidyl glutarate to cross-link proteins to DNA for 15 min at room temperature, followed by sonicating to generate DNA fragments with the average size below 1000 base pairs by lengths. DNA-protein complexes were then immunoprecipitated with 2 μg indicated antibodies or 2 μg anti-IgG antibody as a negative control. The purified DNAs were subjected to conventional PCR analysis following reverse cross-linking of protein-DNA complexes after incubation with 50% slurry of protein A-agarose/salmon sperm DNA (Upstate Biotechnology, Lake placid, NY) for 3 h at 4 °C. The primers used for PCR in the enrichment of PKM2 HRE region were forward: 5′-CTGGAGCGGGAGCGCGAGG-3′ and reverse: 5′-AAGACGGGGTTTCGCCAC G-3′.

### Dual-luciferase reporter assay

Dual-luciferase reporter assays were determined by Dual Luciferase Reporter Assay Kit (Promega, USA) according to the manufacturerʼs recommendation. In brief, The cells underwent variable treaments were transfected with 100 ng of PKM2 promoter luciferase reporter plasmids and 1 ng of pRL-TK Renilla plasmid using Lipofectamine 2000 reagent. The cells were harvested and their luciferase activities were measured forty-eight hours later. Firefly luciferase activity was normalized to Renilla luciferase activity and three independent experiments were performed.

### Real-time quantitative PCR (RT-qPCR)

RT-qPCR experiments were performed as previously indicated^46,47^. Briefly, total RNA was extracted from the indicated PDAC cells using Trizol (Invitrogen) and reverse-transcribed to complementary DNA with PrimeScript^®^RT reagent Kit (Takana, Dalian, China) using Super Array PCR master mix (SuperArray Bioscience, Frederick, Maryland, USA). Real-time PCR was then performed on each sample with the double-stranded DNA dye SYBR Green PCR Mastermix in Takana SYBR^®^ Primix Ex Taq^TM^Kit (Takana, Dalian, China) with the following primers: PKM2 sense, 5′-ATGTCGAAGCCCCATAGTGAA-3′ and PKM2 antisense, 5′-TGGGTGGTGAATCAATGTCCA-3′. The levels of mRNA expression are defined based on Ct and data are presented as mean ± s.d. of five independent experiments.

### Measurements of glucose consumption and lactate production

The indicated PDAC cells were seeded in culture dishes and the conditioned medium was collected for measurement of glucose and lactate concentrations after 24 h using glucose assay kit and lactate assay kit from BioVision (Milpitas, CA, USA). The glucose consumption and lactate production were then normalized to cell number.

### Colony formation assays

Colony formation assays were carried out as previously described^46^. In brief, The indicated cells plated in six-well plate at a density of 1000 cells per well were underwent hypoxia treament and allowed to grow in complete culture medium containing 10% FBS. Ten days later, the colonies were fixed with 4% paraformaldehyde for 5 min, stained with 1% crystal violet for 10 min and rinsed with phosphate-buffered saline (PBS) for three times, photographed and counted.

### Xenograft study

All animal care and experiments were conducted with the approval of the Institutional Animal Care and Use Committee (IACUC) guidance of Huazhong University of Science and Technology (HUST). To establish xenograft models, we subcutaneously injected the indicated PDAC cells (1 × 10^7^) suspended in 200 µL PBS into the right flank of 4-week-old to 6-week-old BALB/c athymic nude mice (*nu/nu*, male) as described in previous publications^46^. From seven days posttransplantation, the tumor volume was measured every 2 days by using a caliper and calculated as length × width^2^/2. Twenty-three days after inoculation, mice were killed by cervical dislocation and xenografts were excised, fixed, weighed, photographed, and stored. Tumor tissues were then embedded by paraffin wax and cut in slices, followed by haematoxylin and eosin (H&E) and IHC staining.

### Immunohistochemistry

All clinical specimens were collected in accordance with the policy of internal review and ethics boards at the hospital and prior patient consent and approval from the Institutional Research Ethics Committee of Sun Yat-Sen University (SYSU) were obtained for the use of these clinical materials for research purposes.

A total of 87 paraffin-embedded PDAC specimens were obtained from Department of Pathology at the First Affiliated Hospital of Sun Yat-Sen University (SYSU) from 2007 to 2009. IHC analyses were performed on the formalin-fixed and paraffin-embedded 5 µm tissue sections as previously described^46^. Following deparaffinization with dimethylbenzene, rehydration with alcohol and antigen retrieval, sections were incubated with the primary antibodies at 4 °C overnight in a humidified container. A Dako ChemMate^TM^ Envision^TM^ Detetcion Kit (DaKo, Glostrup, Denmark) was then used to detect the primary antibodies and the tissue slides were counterstained with hematoxylin. The images were captured using Nikon Ti-S microscope equipped with a digital camera system (Nikon, Japan) and the scores were determined by combining the proportion of positively-stained tumor cells and the staining intensity. The staining were evaluated and scored independently by two experienced observers who were blinded to the data.

### Three-dimensional structure of ERK2-14-3-3ζ docking complex

The ERK2-14-3-3ζ docking complex was generated using the HADDOCK program (version 2.2). The primary structures for docking is the crystal structure of human ERK2 complexed with a MAPK docking peptide (PDB ID: 4H3Q) with a resolution 2.2 Å and X-ray crystal structure of 14-3-3ζ with a resolution 2.7 Å (PDB ID: 1IB1). The ambiguous interaction restraints (AIRs) protocol was performed to generate the structure of ERK2 MAPK grooves binding to 14-3-3ζ D-site. The active residues for ERK2 included L157, N158, T159, Y316, D318, P319, D321 sets and the active residues for 14-3-3ζ included R91, L98, and L100 sets. The neighboring amino acids of the active residues in both proteins were defined as passive residues. More than 10,000 configurations were calculated at first iteration on the basis of rigid body energy minimization. After that, they were allowed to undergo a second iteration with semiflexible simulated annealing by cluster-structural analysis and the best one was selected as a final model according to HADDOCK score.

### Statistical analysis

SigmaStat Statistical Software 17.0 (SPSS Inc, Chicago, IL, USA) was used for all statistical analyses and data were expressed as mean ± standard deviation of at least three independent experiments. Differences between two groups were assessed by an unpaired, two-tailed Student’s *t* test. Bonferroni post-hoc *t* tests were performed to make statistical comparisons in multigroup analysis after a significant result was obtained using ANOVA. Bivariate correlations between study variables were calculated by Spearman’s rank correlation coeffcients. *P* value of <0.05 in all cases was considered statistically significant.

## Supplementary information

Supplemental Figure legends

Supplemental Figure S1

Supplemental Figure S2

Supplemental Figure S3

Supplemental Figure S4

Supplemental Figure S5
