# Supplementary material for: Phosphorylation of 14-3-3ζ links YAP transcriptional activation to hypoxic glycolysis for tumorigenesis
Source: Oncogenesis. 2019 May 10;8(5):31. doi: 10.1038/s41389-019-0143-1 (PMC6510816; doi:10.1038/s41389-019-0143-1)
Supplement: Supplementary file 1 — Supplemental Figure legends [file 41389_2019_143_MOESM1_ESM.pdf]

**Phosphorylation of 14-3-3ζ links YAP transactivation to hypoxic glycolysis for tumorigenesis**

Yu Jia[1+], Hui-Yan Li[2+], Jue Wang[3], Ying Wang[4], Peng Zhang[5], Ning Ma[2], Shi-Jing Mo[2*]

**Supplemental Information**

Page 1-2: Supplemental Fig. Legends S1-S5

Page 3-4: Supplemental Table S1-S2

**Fig. S1 Hypoxia induces nuclear translocation of YAP in a HIF-1α-independent manner. a** Subcellular fractionation analyses determining abundance of nuclear and cytoplasmic YAP protein in A498 RCC cells (A-4) and Huh-7 HCC cells (H-7) stimulated with hypoxia for 6h. GAPDH and Lamin B were used as internal control of cytoplasmic and nuclear extractions, respectively. Data are expressed as mean ± s.d. of three independent experiments. $*P < 0.05$, $**P < 0.01$. Two-sided Student's t test was used to calculate the $P$ value. **b** Subcellular fractionation analyses comparing levels of nuclear YAP protein expression in SW-1990 PDAC cells stimulated with the indicated concentrations of $CoCl_2$ for 12h. **c** Subcellular fractionation analyses comparing accumulation of nuclear and cytoplasmic YAP protein in hypoxia-stimulated SW-1990 PDAC cells with or without HIF-1α siRNA transfection. **d** Coimmunoprecipitation assay evaluating the interaction between Myc-tagged wild-type TEAD4 and nuclear YAP in SW-1990 PDAC cells stimulated with hypoxia for 12h.

**Fig. S2 Hypoxia induces nuclear translocation of YAP in a 14-3-3ζ-dependent fashion. a** Subcellular fractionation analyses assessing abundance of nuclear and cytoplasmic YAP protein expression in SW-1990 PDAC cells with or without 14-3-3ζ siRNA transfection. Data are expressed as mean ± s.d. of three independent experiments. $*P < 0.05$. Two-sided Student's t test was used to calculate the $P$ value. **b** Left panel: Western-blotting comparing the levels of Flag expression in SW-1990 PDAC cells with or without Flag-tagged wild-type 14-3-3ζ transfection. Right panel: Coimmunoprecipitation assay evaluating the interaction between Myc-TEAD4 and nuclear YAP in SW-1990 PDAC cells stimulated with hypoxia for 12h with or without Flag-tagged wild-type 14-3-3ζ transfection. IP, immunoprecipitation. **c** Coimmunoprecipitation assay comparing the interaction between 14-3-3ζ and YAP in SW-1990 PDAC cells treated with or without 1 mmol/L $CoCl_2$ for 12h. Data are expressed as mean ± s.d. of three independent experiments. $*P < 0.05$. Two-sided ANOVA with Bonferroni post hoc t test correction was used to calculate the $P$ value. **d** Coimmunoprecipitation assay assessing the interaction between endogenous WWTR1 and YAP in SW-1990 PDAC cells stimulated with or without hypoxia for 12h.

**Fig. S3 ERK1/2 is required for the hypoxia-stimulated YAP nuclear localization. a** Western-blotting comparing the levels of p-IκBα and p-ERK1/2 in SW-1990 PDAC cells stimulated with hypoxia in the presence or absence of 5 μmol/L BAY 11-7085 (BAY) and 20 μmol/L U0126 pretreatment, respectively. **b** Left panel: Western-blotting testing the levels of ERK2 expression in SW-1990 PDAC cells transfected with ERK2 siRNA. Right panel: Coimmunoprecipitation assay determining the interaction between Myc-tagged wild-type TEAD4 and nuclear YAP in SW-1990 PDAC cells with or without serum stimuli (SS) in the presence or absence of ERK2 siRNA transfection. **c** Alignment of the highly conserved Ser37 residue of ERK2 phosphorylation in 14-3-3ζ from homo sapiens to drosophila.

**Fig. S4 Hypoxia phosphorylates 14-3-3ζ at Ser37 via ERK2. a** Coimmunoprecipitation assay detecting the levels of Flag-tagged wild-type 14-3-3ζ phosphorylation in hypoxia-stimulated SW-1990 PDAC cells in the presence or absence of 5 μmol/L BAY 11-7085 (BAY) and 20 μmol/L U0126 administration with an anti-phospho-serine antibody, respectively. **b** Sequencing analysis of Ser37 mutations in 14-3-3ζ protein. **c** *Top panel*: Co-IP assay testing the specificity of 14-3-3ζ

pS37 antibody. *Bottom panel*: Representative immunfluorescence images testing the specificity of 14-3-3ζ pS37 antibody in SW-1990 PDAC cells with Flag-tagged wild-type 14-3-3ζ (WT) or mutant 14-3-3ζ Ser37A (S37A) expression. Scale bar = 25 μm. **d** RT-qPCR analyses of YWHAZ gene expression in 14-3-3ζ shRNA (sh. 14-3-3ζ)-expressed SW-1990 PDAC cells. Experiments were performed five times, each with quantitative RT-PCR in technical duplicate and real-time values were normalized to glyceraldehyde 3-phosphate dehydrogenase (GAPDH). Data are expressed as mean ± s.d. ***$P < 0.001$. Two-sided Student's t test was used to calculate the *P* value.

**Fig. S5 YAP facilitates PKM2 transcription via physical interaction with HIF-1α in an 14-3-3ζ Ser37 phosphorylation-dependent manner under hypoxia. a** Coimmunoprecipitation assay examining the interaction between nuclear YAP and HIF-1α in hypoxia-stimulated SW-1990 PDAC cells with or without ERK2 siRNA transfection. **b** Coimmunoprecipitation assay determining the interaction between nuclear YAP and HIF-1α in 14-3-3ζ shRNA (sh.14-3-3ζ)-expressed SW-1990 PDAC cells in the presence or absence of Flag-tagged wild-type 14-3-3ζ (WT) or mutant 14-3-3ζ S37E (S37E) reconstitution. **c** Top panel: Western blotting results depicting the expression levels of YAP with scrambled shRNA (Scr) or YAP shRNA transfection. Bottom panel: ChIP analysis for HIF-1α and HIF-2α binding to PKM2 gene HRE in SW-1990 PDAC cells stimulated with hypoxia in the presence or absence of YAP shRNA transfection using the indicated antibodies. **d** Top panel: Western blotting results depicting the expression levels of HIF-1α with control siRNA (Ctrl) or HIF-1α siRNA transfection. Bottom panel: ChIP analysis for YAP binding to PKM2 gene HRE in SW-1990 PDAC cells stimulated with hypoxia in the presence or absence of HIF-1α siRNA transfection using an anti-YAP antibody. **e** RT-qPCR analyses of PKM2 gene expression in YAP shRNA-expressed SW-1990 PDAC cells with hypoxia stimulation. Experiments were performed five times, each with quantitative RT-PCR in technical duplicate and real-time values were normalized to glyceraldehyde 3-phosphate dehydrogenase (GAPDH). Data are expressed as mean ± s.d. *$P < 0.05$. Two-sided Student's t test was used to calculate the P value. **f** Western-blotting measuring levels of PKM2 protein expression in YAP shRNA-expressed SW-1990 PDAC cells with hypoxia stimulation. **g** Representative immunfluorescence images of Flag-tagged 14-3-3ζ staining in 14-3-3ζ shRNA (sh.14-3-3ζ)-expressed SW-1990 PDAC cells with Flag-tagged wild-type 14-3-3ζ (WT) or mutant 14-3-3ζ Ser37A (S37A) reconstitution. Scale bar = 100 μm. **h** Immunohistochemistry analysis of 14-3-3ζ p-S37 antibody with or without 14-3-3ζ peptide used for developing this antibody or the matched nonphosphorylated 14-3-3ζ peptide. Scale bar = 50 μm. **i** Representative cases stained by immunohistochemistry (*left panel*) and bar graph (*right panel*) showing the expression of 14-3-3ζ p-S37 in 56 primary human PDAC specimens is positively correlated with nuclear localization of YAP. Scale bar = 50 μm. The *P* value shown was calculated by Spearman order correlations.

**Table S1 Clinicopathological Features in PDAC Patients (n=87)**

| Variable | No. of patients | % |
|---|---|---|
| Gender | | |
| Male | 50 | 57.5 |
| Female | 37 | 42.5 |
| Age (years) | | |
| <60 | 33 | 37.9 |
| ≥60 | 54 | 62.1 |
| Tumor size | | |
| ≤40mm | 56 | 64.4 |
| >40mm | 31 | 35.6 |
| Tumor location | | |
| Head | 71 | 81.6 |
| Body | 9 | 10.3 |
| Tail | 7 | 8.1 |
| Lymphatic metastasis | | |
| Negative | 49 | 56.3 |
| Positive | 38 | 43.7 |
| TNM stage | | |
| I | 22 | 25.3 |
| II | 43 | 49.4 |
| III | 14 | 16.1 |
| IV | 8 | 9.2 |
| Chemotherapy | | |
| Yes | 72 | 82.8 |
| No | 15 | 17.2 |
| p-14-3-3ζ expression | | |
| High | 45 | 51.7 |
| Low | 42 | 48.3 |

**Table S2 Correlation Between Phosphorylation of 14-3-3ζ and Clinicopathological Features in PDAC Patients**

| Variable | p-14-3-3ζ High (n = 45) | p-14-3-3ζ Low (n = 42) | *P* value |
|---|---|---|---|
| Gender | | | 0.313 |
| Male | 27 (60.0%) | 23 (54.8%) | |
| Female | 18 (40.0%) | 19 (45.2%) | |
| Age (years) | | | **0.015** |
| <60 | 22 (48.9%) | 11 (26.2%) | |
| ≥60 | 23 (51.1%) | 31 (73.8%) | |
| Tumor size | | | < **0.001** |
| ≤40mm | 20 (44.4%) | 36 (85.7%) | |
| >40mm | 25 (55.6%) | 6 (14.3%) | |
| Tumor location | | | 0.103 |
| Head | 37 (82.2%) | 34 (81.0%) | |
| Body | 6 (13.3%) | 3 (7.1%) | |
| Tail | 2 (4.5%) | 5 (11.9%) | |
| Lymphatic metastasis | | | **0.031** |
| Negative | 21 (46.7%) | 28 (66.7%) | |
| Positive | 24 (53.3%) | 14 (33.3%) | |
| TNM stage | | | 0.200 |
| I | 14 (31.1%) | 8 (19.0%) | |
| II | 17 (37.8%) | 26 (61.9%) | |
| III | 8 (17.8%) | 6 (14.3%) | |
| IV | 6 (13.3%) | 2 (4.8%) | |
| Chemotherapy | | | 0.104 |
| Yes | 35 (77.8%) | 37 (88.1%) | |
| No | 10 (22.2%) | 5 (11.9%) | |
