# Supplementary figures and images for: Phosphorylation of 14-3-3ζ links YAP transcriptional activation to hypoxic glycolysis for tumorigenesis
Source: Oncogenesis. 2019 May 10;8(5):31. doi: 10.1038/s41389-019-0143-1 (PMC6510816; doi:10.1038/s41389-019-0143-1)

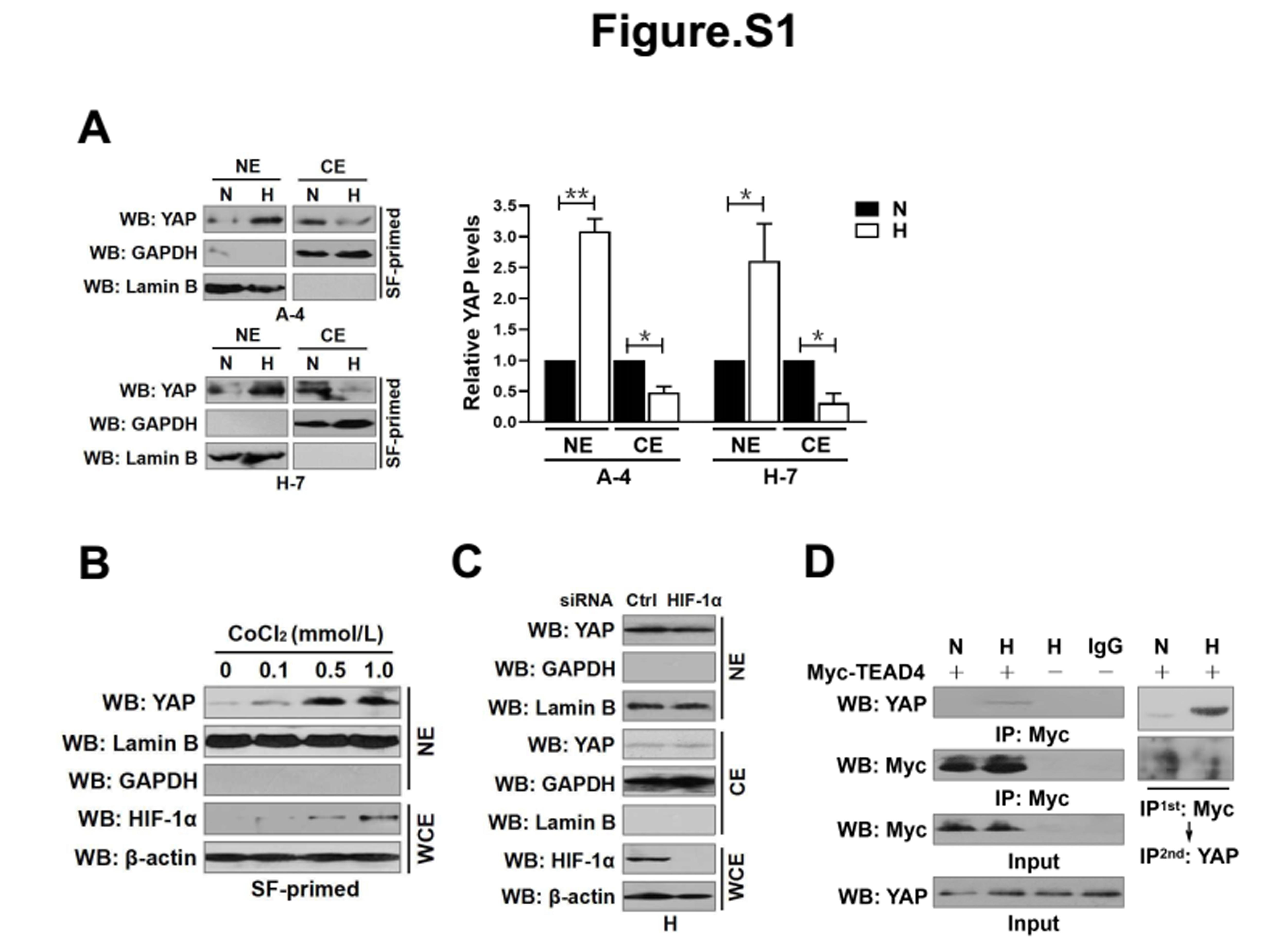

Supplement: Supplementary file 2 — Supplemental Figure S1 [file 41389_2019_143_MOESM2_ESM.tif]

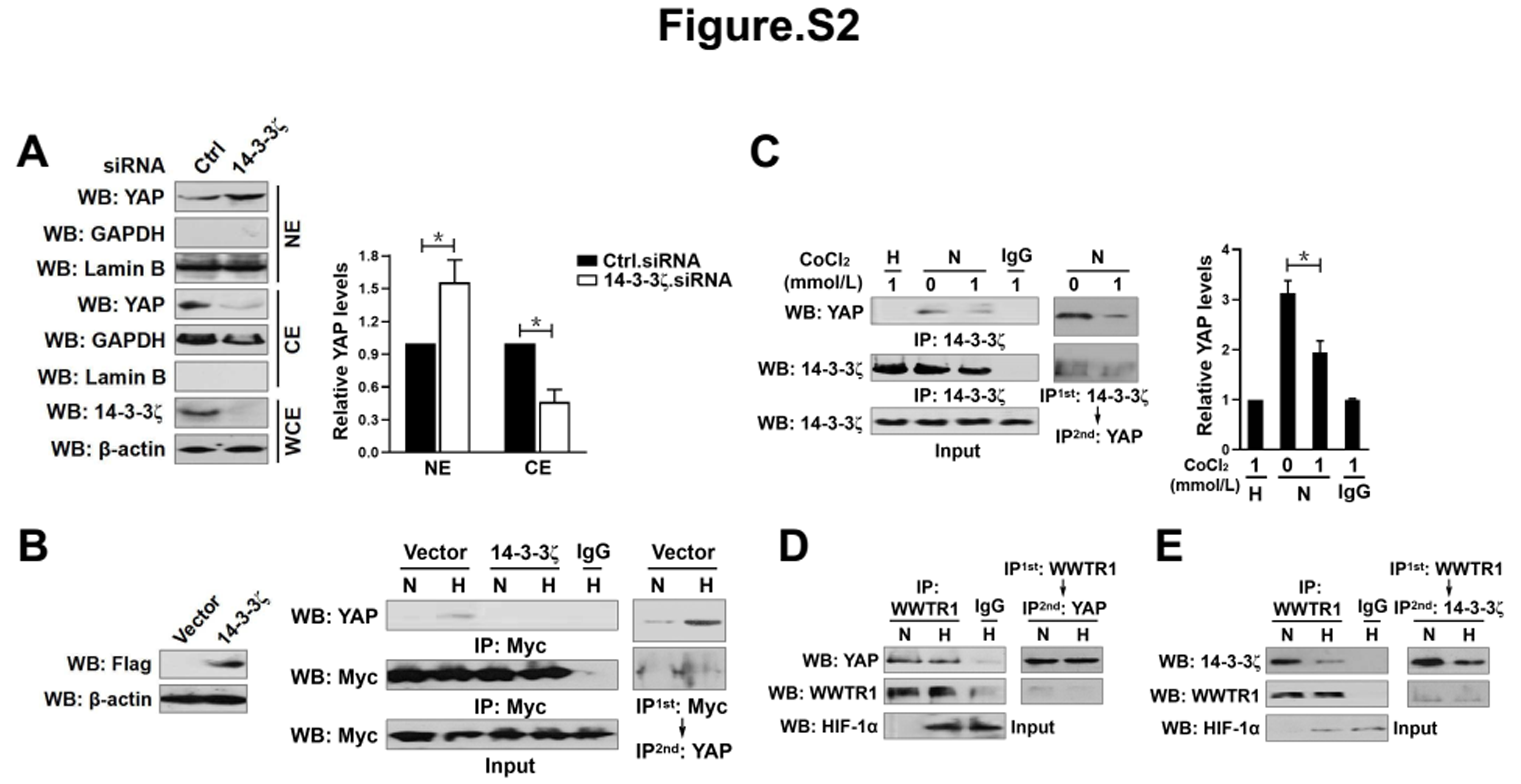

Supplement: Supplementary file 3 — Supplemental Figure S2 [file 41389_2019_143_MOESM3_ESM.tif]

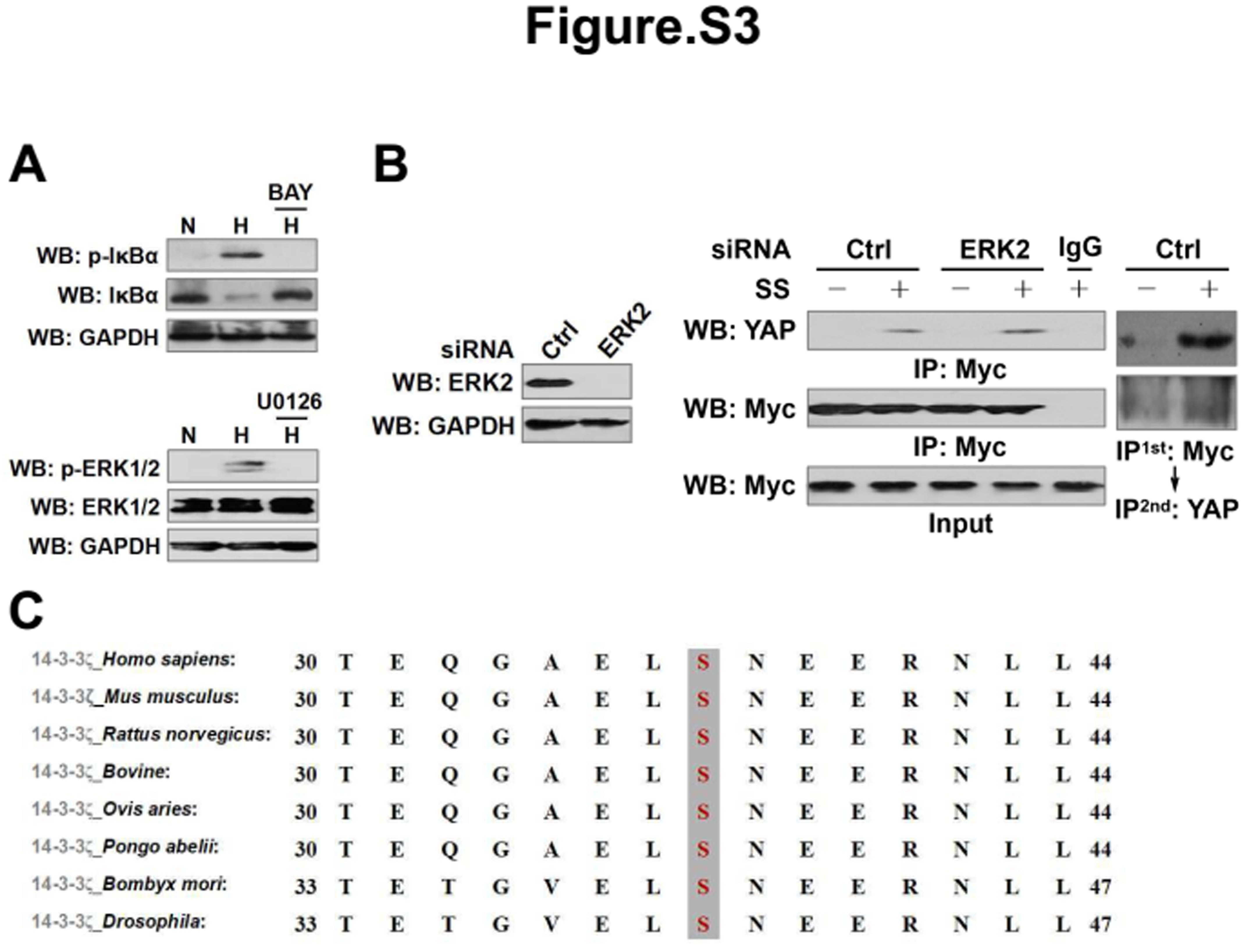

Supplement: Supplementary file 4 — Supplemental Figure S3 [file 41389_2019_143_MOESM4_ESM.tif]

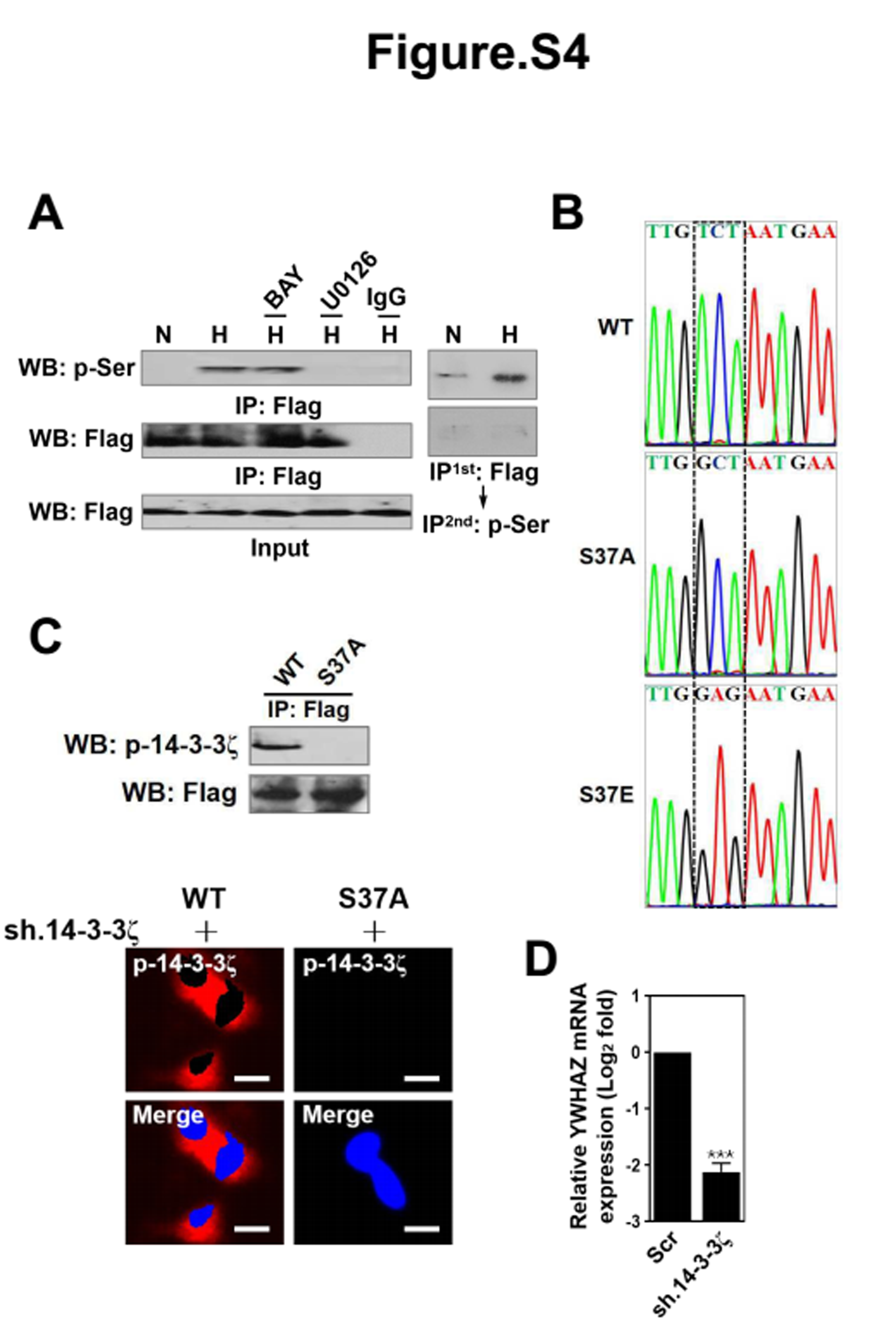

Supplement: Supplementary file 5 — Supplemental Figure S4 [file 41389_2019_143_MOESM5_ESM.tif]
